# Multiplexed Fluorescent Microarrays on MIL‐101(Cr) Thin Films as Luminescent Probes for pH and Disease‐Associated Molecules

**DOI:** 10.1002/smll.202504783

**Published:** 2025-09-10

**Authors:** Wenjing Wang, Wenwu Yang, Maike Schliephake, Tonghan Zhao, Yan Liu, Navid Hussain, Ben Breitung, Andreas H. Schäfer, Pavel A. Levkin, Jasmin Aghassi‐Hagmann, Annie K. Powell, Michael Hirtz

**Affiliations:** ^1^ Institute of Nanotechnology (INT) Karlsruhe Institute of Technology (KIT) Kaiserstraße 12 76131 Karlsruhe Germany; ^2^ Karlsruhe Nano Micro Facility (KNMFi) Karlsruhe Institute of Technology (KIT) Kaiserstraße 12 76131 Karlsruhe Germany; ^3^ Institute of Biological and Chemical Systems (IBCS‐FMS) Karlsruhe Institute of Technology (KIT) Kaiserstraße 12 76131 Karlsruhe Germany; ^4^ Institute of Microstructure Technology (IMT) Karlsruhe Institute of Technology (KIT) Kaiserstraße 12 76131 Karlsruhe Germany; ^5^ Institute of Inorganic Chemistry (AOC) & Institute for Quantum Materials and Technologies (IQMT) Karlsruhe Institute of Technology (KIT) Kaiserstraße 12 76131 Karlsruhe Germany; ^6^ nanoAnalytics GmbH Heisenbergstraße 11 48149 Münster Germany

**Keywords:** fluorescent chemosensors, metal‐organic frameworks, principal component analysis, scanning probe lithography

## Abstract

Recently, metal‐organic frameworks (MOFs) have shown high potential in the field of sensing. However, fluorescent‐based detection with MOFs in solution needs complex pre‐treatments and has stability issues, complicating measurements and handling for sensing applications. Here, an easy‐to‐handle and low‐cost strategy is introduced to convert MOF‐based sensing from solution to surface using scanning probe lithography. The MOF is immobilized on the surface by receding meniscus coating, and then fluorescent dyes are patterned on the MOF thin films through microchannel cantilever spotting to generate dye@MOF fluorescent microarrays, which are stable in analyte solutions. The dye@MOF fluorescent microarrays exhibit good pH sensitivity in the pH 5–9 range, and dopamine can be distinguished from three other metabolites in the solution by these microarrays when signals from different dyes are analyzed in combination with principal component analysis. This concept provides a new approach for stable microarray‐based detection of small molecule analytes from a fluid environment.

## Graphical abstract

In this study, a receding meniscus coating method is proposed for the fabrication of MOF thin film with good morphology, and multiplexed fluorescent microarrays based on four common dyes are developed on MIL‐101 thin films. The multiplexed fluorescent microarrays exhibit good stability in solution, pH sensitivity, and potential for discriminating dopamine from other interferents.

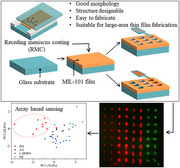

## 1 Introduction

In recent decades, metal‐organic frameworks (MOFs) have been demonstrated as a highly promising material for various applications due to their potential in gas storage/separation, catalysis, analytical chemistry, electronic and ionic conduction, and magnetic applications.^[^ ^1^, ^2^ ^]^ With their tunable pore sizes and structural versatility, MOFs offer specific selectivity and sensitivity toward various molecules, which other materials can hardly achieve.^[^ ^3^, ^4^, ^5^ ^]^ Leveraging these structural advantages over conventional porous materials, researchers have developed MOF‐based sensing platforms by optimizing pore size and shape to enhance guest molecule adsorption; functionalizing the framework with specific chemical groups to enable selective analyte interaction; and tuning metal node coordination to tailor electronic properties.^[^ ^6^, ^7^ ^]^ For example, Herm et al. synthesized a highly stable Fe‐based MOF Fe_2_(BDP)_3_ with the absorption selectivity of hexane isomers due to its unique triangular channel.^[^ ^8^ ^]^ The size of the channel in Fe_2_(BDP)_3_ MOF is sufficiently large to accommodate the selected hexane isomers, and the sharply angled pore walls enhance the adsorption for linear alkanes via the van der Waals contact, which is more than the adsorption of branched ones, making the Fe_2_(BDP)_3_ more effective than the traditional materials like zeolite that separate the hexane isomers based on the size selectivity. Therefore, MOF‐based detection platforms have become increasingly popular, particularly for the MOF‐based fluorescent chemosensors that dyes are introduced into as indicators due to their simplicity of synthesis and the possibility of tuning the emission properties by varying the choice of dyes.^[^ ^9^ ^]^ The intrinsic porosity and highly ordered structure of MOFs provide a confined environment for hosting luminescent guest molecules, which strengthens analyte interactions and thereby improves sensing performance.^[^ ^10^ ^]^

However, most MOF‐based fluorescent chemosensors work in a liquid environment and require proper and careful preparation to prevent introducing significant errors. For example, a thorough sonication treatment to form a stable suspension or emulsion before use is critical to prevent potential errors that could arise if there are some concentrated sites facing the light source that absorb more incident light at the surface.^[^ ^11^ ^]^ Moreover, the particles that drift through the laser beam can lead to intensity fluctuations.^[^ ^11^ ^]^ Transferring the fluorescent detection process from the solution to a surface can eliminate the preparation step in the solution and decrease the possibility of errors caused by turbidity. Hence, developing MOF thin films with fluorescence is a better alternative for detection in solution, and the methods that can provide good structural control of MOF thin films and be applied on a large area are desired for the sensing application on the surface.

On the other hand, the amount of synthetic fluorescent dye used in the fabrication of dye@MOF chemosensor needs to be considered because a lot of common organic dyes, such as rhodamine B and methylene blue etc., can become toxic contaminants in water without scientific treatment.^[^ ^12^ ^]^ Scanning probe lithography (SPL) based techniques offer the ability to directly pattern dye on a large variety of materials with nanoscale precision in feature size and position, reducing the use of dye solution to the femtoliter scale.^[^ ^13^ ^]^ The dye solution penetrates into the MOF thin film via SPL to form a dye@MOF fluorescent spot, which endows surfaces with a potential fluorescent sensing function. A microarray of fluorescent spots also can be obtained readily by SPL. Every spot in the microarray is independent of the others, allowing the results of a collection of repeat measurements on the sample to be obtained in a single step. This is a more rapid process than the measurement in the cuvette, which only produces data one at a time. Additionally, SPL methods can integrate several indicators with different structures or emission properties into one substrate to form an array‐based sensor, which offers a parallel readout of multiple sensing elements arranged in a systematic configuration. When combined with principal component analysis (PCA), this approach improves the overall selectivity of MOF‐based fluorescent chemosensors, which only show partial selectivity depending on non‐specific interactions between binding partners or the shape and size selectivity of the binding cavities that are unable to distinguish the analytes from other interference with similar chemical structures, thereby overcoming the limitations of traditional sensing techniques in complex environments.^[^ ^14^ ^]^

Based on the above reasons, we developed a multiplexed array‐based sensing system by patterning four red and green dye indicators with different structures on the MIL‐101(Cr)‐NH_2_ thin film via the SPL‐based technique of microchannel cantilever spotting (µCS). MIL‐101(Cr) is one of the most studied MOFs with high water stability and chemical stable ability, which has large porosities with 12 Å and 16 Å × 14.5 Å windows (Figure S1, Supporting Information) as well as a high Brunauer‐Emmett‐Teller (BET) surface area (>3000 m^2^/g), making it a promising adsorbent for the fluorescent dyes to make the MOF‐based chemosensors.^[^ ^15^, ^16^ ^]^ Furthermore, the functionalization of MIL‐101(Cr) structure can provide more specific active sites to enhance the interaction between the host and fluorescent dyes. For example, the ‐NH_2_ group on 2‐aminoterephthalic acid ligand can enhance the driving forces of anionic dye adsorption onto MIL‐101(Cr)‐NH_2_, such as the hydrogen binding and electrostatic, to improve the adsorption capacities.^[^ ^17^ ^]^ The MIL‐101 thin films with excellent surface morphology were fabricated on the glass substrate via receding meniscus coating (RMC, Section 2.2). The dyes@MOF composites showed excellent fluorescent intensities under the inverted fluorescence microscope and good stability due to the interaction between the ‐NH_2_ group on the MOF structure and the functional group of dye. FITC@MIL‐101(Cr)‐NH_2_ has pH‐dependent emission, which can serve as the pH indicator. Furthermore, the fluorescent response of dyes@MIL‐101(Cr)‐NH_2_ composites to four neurotransmitters in the fluidic environment was also measured, which demonstrated different fingerprints to analytes and has a potential application for discriminating disease‐associated molecules in solution.

## 2 Results and Discussion

### 2.1 Design and Material Characterization

**Scheme 1:**
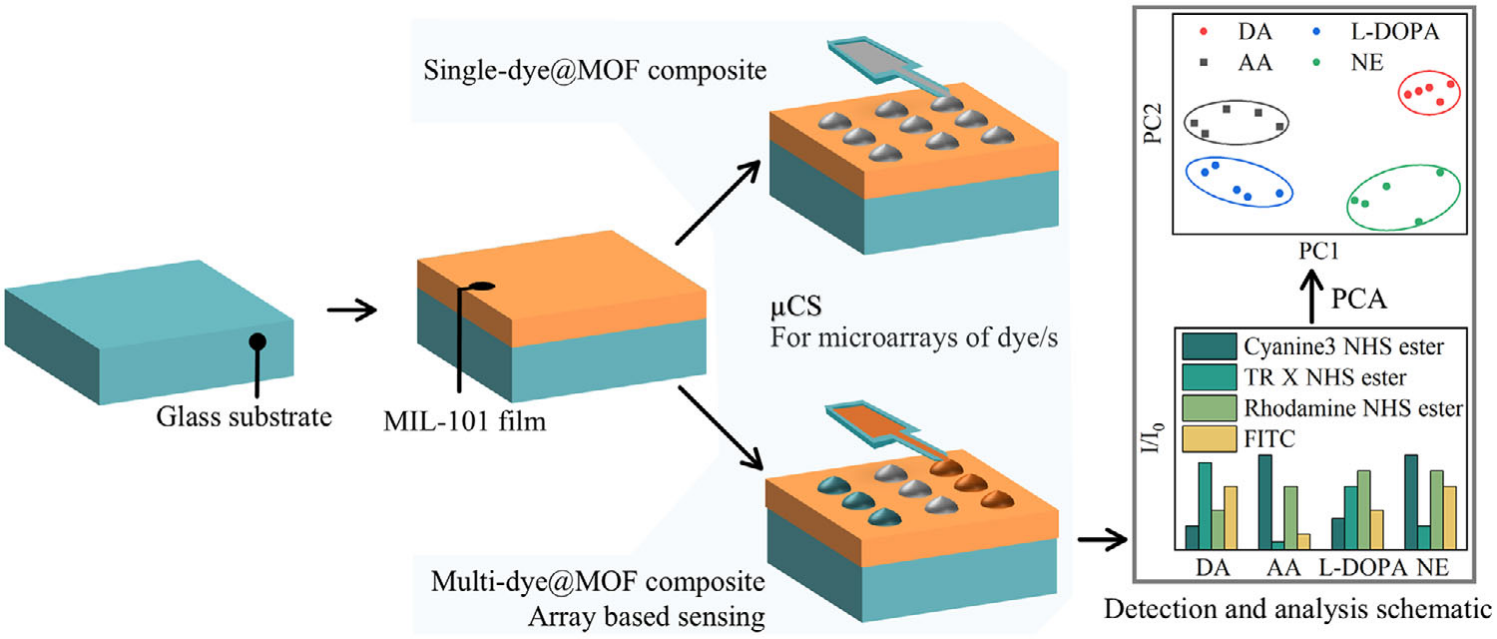
Schematic illustration of the fabrication of the microarrays on MOF thin films via microchannel cantilever spotting (µCS), followed by detection, and data analysis (Conceptional illustration; not actual data).

The design principle is to obtain the microarray‐based fluorescent sensing system in the MOF thin film via the µCS method, which is easy to handle and read out, and can increase the reusability of sensors and decrease material consumption, as shown in **Scheme** **1**. The interaction between analytes and fluorescent probes can alter the optoelectronic properties of fluorescent materials via energy or electron transfer processes, resulting in observable changes in fluorescence intensity or peak shifts in emission spectra.^[^ ^18^ ^]^ This phenomenon is widely regarded as the underlying mechanism of fluorescent sensing. MOFs are promising candidates as supports for fluorescent probes because the nanoscale confinement effect from the MOF pore potentially can improve the efficiency of energy or electron transfer in the host‐guest interaction to facilitate the sensitivity and selectivity of the fluorescent chemosensors.^[^ ^19^, ^20^ ^]^ MIL‐101(Cr) and MIL‐101(Cr)‐NH_2_ were selected for thin film fabrication to serve as substrates for dye immobilization because of their suitable pore size for dye absorption and the possibility of interaction between amino groups and dye functional groups to reduce dye leakage. The patterning of dye on the MOF films was conducted via µCS. Several studies have demonstrated that µCS is an effective technique for permeating droplets into the porous substrate to achieve the desired pattern.^[^ ^21^, ^22^, ^23^ ^]^ In µCS, a microchannel on the surface patterning tool (SPT) tip connects a µL‐scale reservoir to the aperture, allowing continued permeation at a specific site. Since the ink molecule is transferred from tip to the substrate through a meniscus formed when the tip touches the substrate gently, the tip does not damage the MOF film or leave traces. Compared to immobilizing the pre‐synthesized dye@MOF on the surface directly, patterning on the MOF film does not need the synthesis process step of dye@MOF composites in the solution, and also, multi‐dye patterns on one substrate can be readily created to obtain multiplexed array‐based sensors.

**Figure 1:**
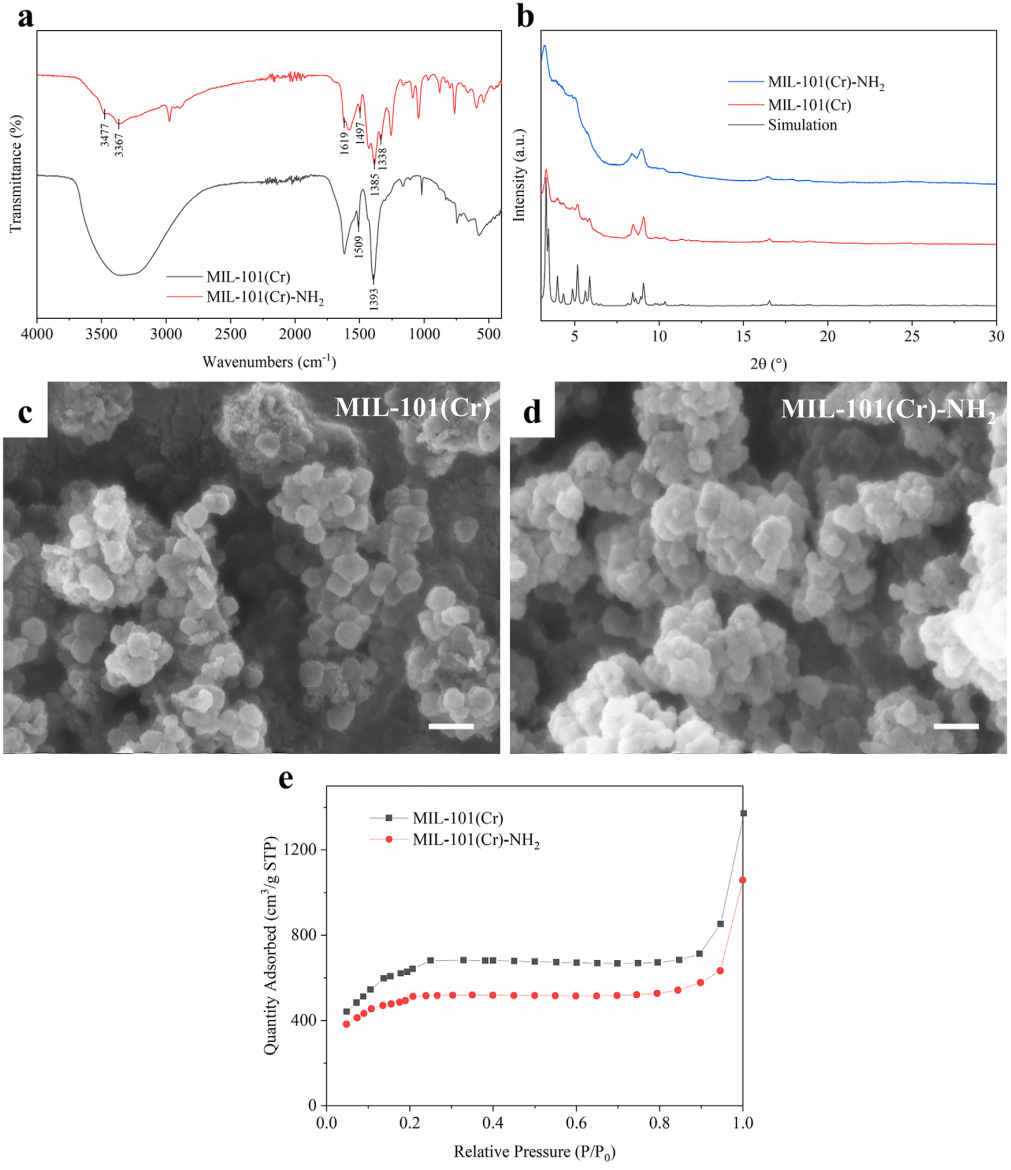
FT‐IR spectra a) and PXRD patterns b) of MIL‐101(Cr) and MIL‐101(Cr)‐NH_2_; SEM image of MIL‐101(Cr) c) and MIL‐101(Cr)‐NH_2_ d), the scale bars represent 100 nm; e) N_2_ adsorption isotherms plots of MIL‐101(Cr) and MIL‐101(Cr)‐NH_2_. P is the gas pressure, and P_0_ is the saturation pressure.

The MIL‐101(Cr) and MIL‐101(Cr)‐NH_2_ were synthesized using the hydrothermal reaction method and verified by FT‐IR and PXRD. **Figure** **1**a shows the FT‐IR spectra of MIL‐101(Cr) and MIL‐101(Cr)‐NH_2,_ respectively. The vibration signals at 1393 and 1509 cm^−1^ (for MIL‐101(Cr)) or 1385 and 1497 cm^−1^ (for MIL‐101(Cr)‐NH_2_) relate to the asymmetric stretching of the ‐(O─C─O)‐, indicating the formation of the MOF framework due to the coordination between the linker and metal ion.^[^ ^24^ ^]^ In the FT‐IR spectrum of MIL‐101(Cr)‐NH_2_, the peak at 1619 cm^−1^ is attributed to the N─H bending vibration of the amino group, while the signal at 1338 cm^−1^ corresponds to the stretching vibration of the C─N bond on the benzene ring.^[^ ^25^ ^]^ The bands at 3477 and 3367 cm^−1^ are ascribed to the asymmetric and symmetric vibration of the ‐NH_2_.^[^ ^26^ ^]^ The PXRD patterns (Figure 1b) indicate the crystal structure of both MIL‐101(Cr) and MIL‐101(Cr)‐NH_2,_ which are highly similar to the simulated pattern but have broader Bragg peaks reflection. Principally, the full width at half maximum of XRD is inversely proportional to the crystallite size, as the crystal gets smaller while the peaks get broader.^[^ ^27^ ^]^ The size and morphology of MIL‐101(Cr) and MIL‐101(Cr)‐NH_2_ were confirmed by SEM, as shown in Figure 1c,d. Both MOF products exhibit multifaceted structures with a diameter of ≈50 nm and approximate spherical shapes due to the nano size. The above results demonstrate the successful synthesis of the nano‐size MIL‐101(Cr) and MIL‐101(Cr)‐NH_2_. The porosity of MOF was characterized by N_2_ absorption, and the absorption isotherm curves of MIL‐101(Cr) and MIIL‐101(Cr)‐NH_2_ are shown in Figure 1e. The BET surface area and total volume of pores of MIL‐101(Cr) are calculated to be 2326.54 m^2^/g and 1.32 cm^3^/g, respectively, in good agreement with values of 2579 m^2^/g and 1.23 cm^3^/g in the literature.^[^ ^28^ ^]^ MIL‐101(Cr)‐NH_2_ presents a lower BET surface area (1746.68 m^2^/g) and total volume of pores (1.04 cm^3^/g) compared to the synthesized MIL‐101(Cr) because of the presence of the ‐NH_2_ group in the organic linker. The adsorption average pore diameter calculated by BET is 2.27 nm in MIL‐101(Cr) and 2.38 nm in MIL‐101(Cr)‐NH_2_, demonstrating the formation of mesoporous cages in the MOF crystal structures.

### 2.2 MOF Film Fabrication

**Figure 2:**
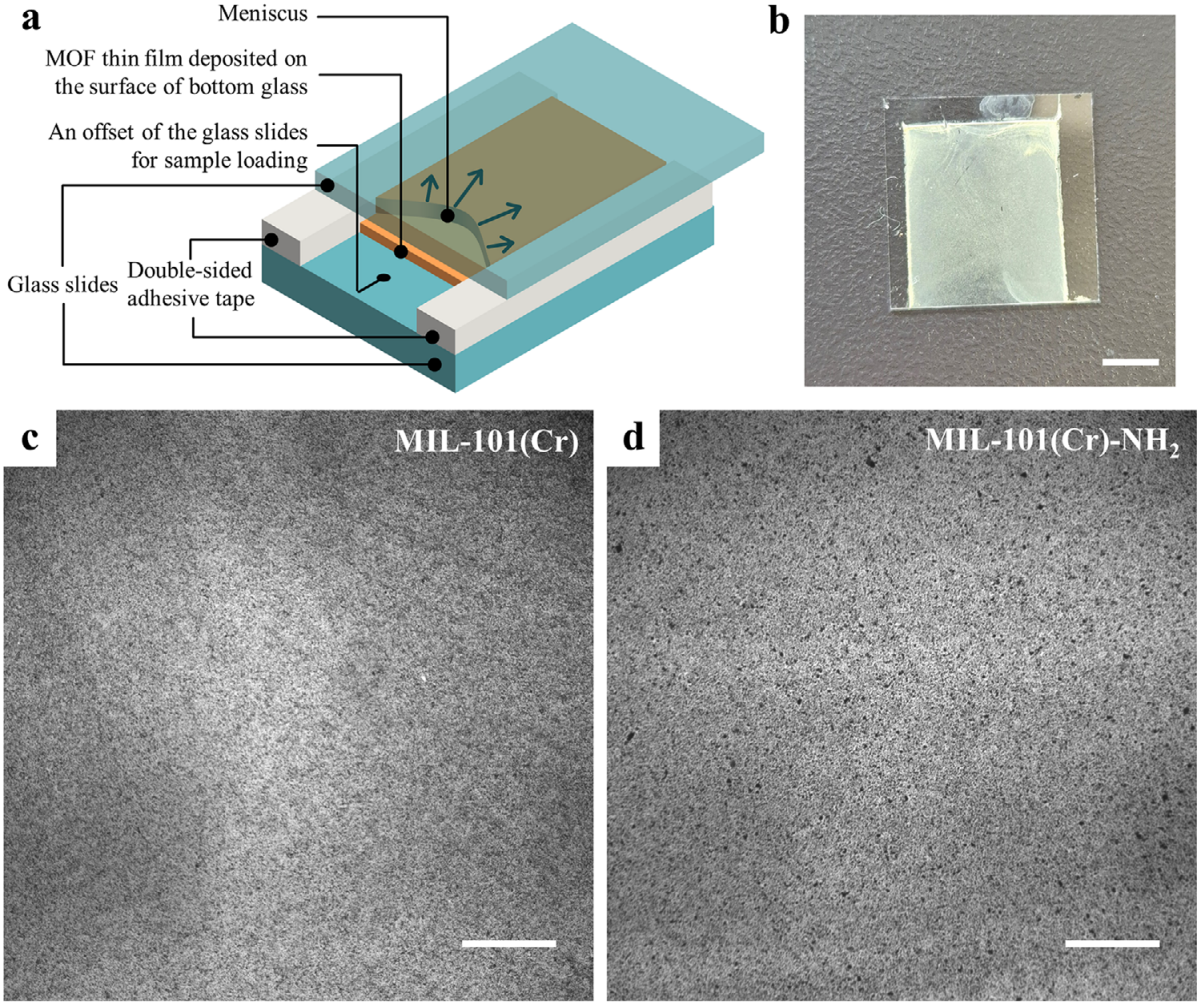
a) Schematic of the receding meniscus coating (RMC) procedure. b) Photograph of MOF film made by RMC. c) Microscope images of MIL‐101(Cr) film and d) MIL‐101(Cr)‐NH_2_ film made by RMC. The scale bars represent 5 mm in (b); and 500 µm in (c) and (d).

A number of techniques have been developed to prepare MOF thin films that can be classified as solution‐based fabrication (liquid‐phase epitaxy, interfacial synthesis, substrate‐seeded heteroepitaxy, electrochemical deposition, and powder MOF‐based deposition) and vacuum‐based fabrication (chemical vapor deposition and atomic layer deposition).^[^ ^29^ ^]^ In addition to the abovementioned methods, Lee et al. introduced a meniscus‐guided crystallization technique to grow high‐quality MOF thin films in a large area for the first time.^[^ ^30^ ^]^ In meniscus‐guided crystallization, the supersaturation precursor solution is sandwiched between the substrate and a moving blade, and the crystallization of MOF occurs in the meniscus to form a thin film. The meniscus‐guided crystallization offers precise control of the crystal packing density, thickness of the film, and crystal size by tuning the experiment parameters, such as the coating speed and substrate temperature, demonstrating it is a facile and powerful technique for various MOF thin film fabrication on different substrates that can overcome the barriers of conventional MOF thin film growth methods.^[^ ^30^ ^]^ In the present study, the RMC technique, modified from meniscus‐guided coating, was employed for the fabrication of MOF thin films for the first time, which is presented in **Figure** **2**a. Here, the suspension of as‐synthesized MOF crystals was used instead of a precursor solution for crystallization during the coating because the formation of MIL‐101 requires harsh conditions such as high temperature and autogenous pressure. Two glass slides were used in RMC, and the bottom one as substrate was treated with oxygen plasma, inducing a high density of ─OH groups on the surface for a good wettability of MOF suspension and immobilization of MOF crystals. The right and left ends of these two glass slides were bonded together with some offset for sample loading by double‐sided adhesive tape to leave a distance of ≈100 µm (the thickness of tape), then the MOF suspension was injected between the two glass slides, and a meniscus formed naturally (Video S1, Supporting Information). The crystals in the meniscus were deposited directly on the substrate to obtain the MOF film. In contrast to the meniscus‐guided crystallization, RMC does not require an actual moving blade because as the solvent evaporated gradually, the liquid edge of the meniscus moved through the entire film, acting like a moving blade to “smooth” the surface and move the excess crystal that could not be deposited on the surface immediately and steadily to the edges or corners (Video S2, Supporting Information). This method allows the fabrication of MOF films with the desired pattern in one step by employing double‐sided tape to create the desired shapes in a specified location, which facilitates obtaining samples that can be readily incorporated with other devices, such as a microfluidic channel system (Figure S2, Supporting Information).

Spin coating and drop casting are commonly used for MOF film fabrication due to their simplicity; however, there are obvious difficulties in controlling morphology, especially for the large substrates, e.g., the so‐called coffee ring effect (the ring‐like structure formed at the edge or contact line when a solution or dispersion dries on a surface, which is caused by the difference in evaporation rate between the edge area and the center) occurs in both spin coating and drop casting, and the film thickness is highly heterogeneous in drop casting.^[^ ^31^ ^]^ However, comparing the photographs and microscope images of MOF thin films made by different deposition methods (Figure 2b–d; Figure S3, Supporting Information), the film made by RMC shows higher flatness than those made by spin coating and drop casting. From the results, there was no coffee ring effect in the RMC, and the capillary force from the meniscus improved the homogeneity of the films, indicating that the RMC is a facile technique with the potential to obtain high‐quality MOF films even superior to spin coating and drop casting.

**Figure 3:**
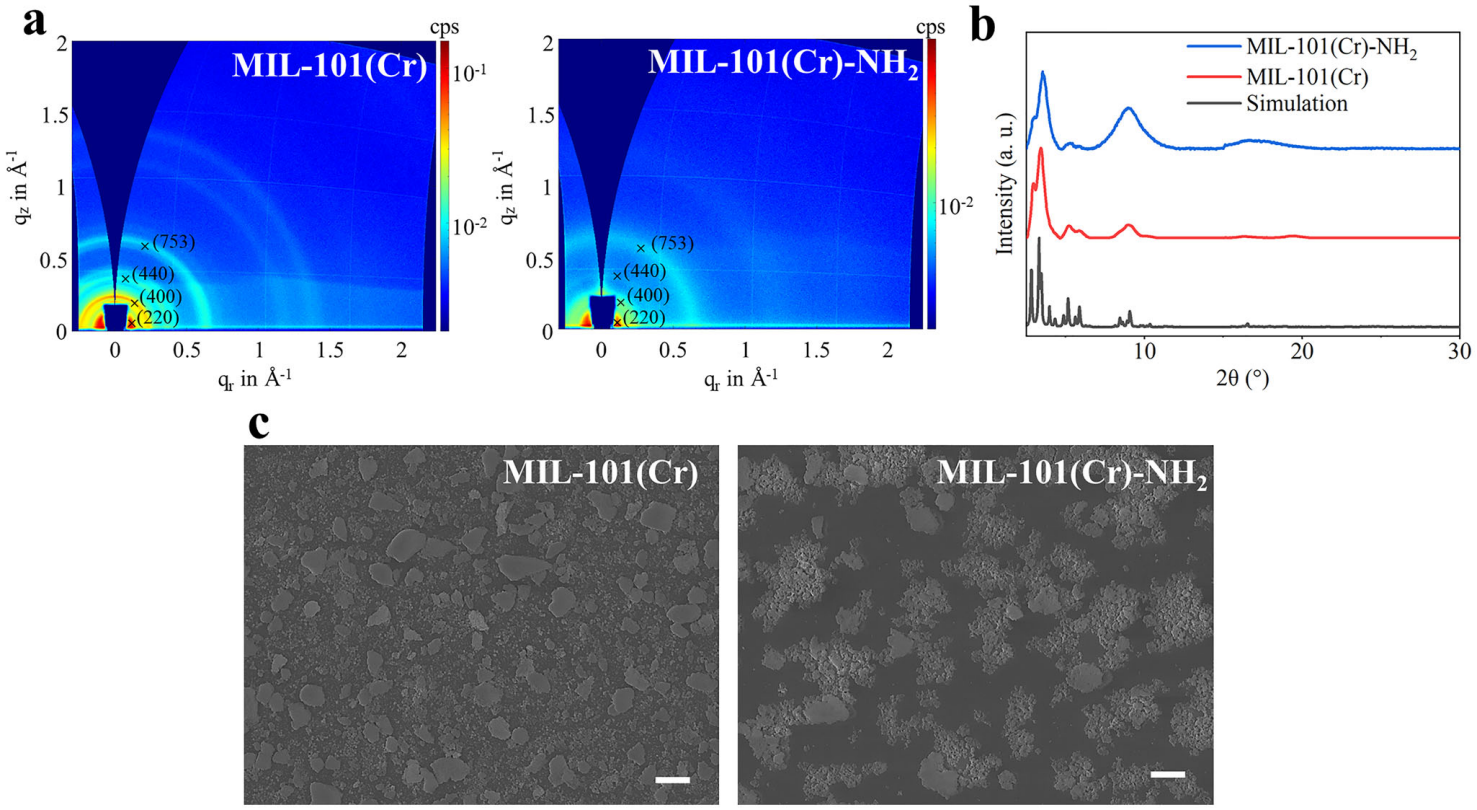
a) 2D GIWAXS patterns of MIL‐101(Cr) and MIL‐101(Cr)‐NH_2_ thin films fabricated via RMC. b) Corresponding XRD patterns obtained by radial integration of the GIWAXS data over all azimuthal angles are shown in (a). c) SEM images of MIL‐101(Cr) film and MIL‐101(Cr)‐NH_2_ film in 2000× amplification. The scale bars represent 4 µm.

The crystallinity of the MOF thin films was evaluated by grazing‐incidence wide‐angle X‐ray scattering (GIWAXS). Since the small amount of crystals deposited on the surface limits the effectiveness of standard PXRD for structural analysis of MOF thin film, GIWAXS, a surface‐sensitive technique, has emerged as an excellent alternative, which effectively overcomes the challenges associated with PXRD and enables reliable crystallinity characterization of MOF thin films.^[^ ^32^ ^]^ **Figure** **3**a presents multi‐frame GIWAXS images of MOF thin films fabricated via RMC, capturing the 2D diffractogram at various scattering vector *q* values. Distinct diffraction rings are observed in images, with prominent features at *q*≈0.64, 0.44, 0.28, and 0.20 Å^−1^, corresponding to the (753), (440), (400), and (220) crystal planes, respectively, which are in good agreement with the reported structure of MIL‐101.^[^ ^24^, ^33^ ^]^ The XRD patterns derived from the GIWAXS data (Figure 3b) are also highly consistent with the simulated PXRD patterns of MIL‐101, although the characteristic peaks appear broader. This broadening is likely due to the limited amount of material deposited on the substrate, as well as the intrinsic peak broadening inherent to GIWAXS analysis.^[^ ^34^ ^]^ These results indicate that the crystal structures of MIL‐101(Cr) and MIL‐101(Cr)‐NH_2_ are well preserved after thin film fabrication. The surface morphologies of MOF films made by RMC were characterized by SEM, shown in Figure 3c and Figure S4 (Supporting Information). According to Figure S4a,b (Supporting Information), the two MOFs retained their original structures after the fabrication of films, as observed in the synthesized crystals shown in Figure 2c,d. The MIL‐101(Cr) film exhibited a denser and more uniform particle distribution compared to MIL‐101(Cr)‐NH_2_, with no large voids, while empty spaces with sizes up to several micrometers were observed in the MIL‐101(Cr)‐NH_2_ film (Figure 3), which may be attributed to the agglomeration of crystals occurring due to the amine functional group on the MOF framework.^[^ ^35^, ^36^ ^]^ The roughness and thickness of MOF films were evaluated using a 3D optical profilometer, which applies an optical probe to characterize the surface morphologies by sensing the optimal focus position on the surface rather than a mechanical probe that contacts the test surface. The 3D surface images of the MOF film (Figure S5, Supporting Information) were generated from the collection of height data at every single point on the surface, and the root mean square heights of the whole field were calculated as 3.28 µm for MIL‐101(Cr) film and 3.57 µm for MIL‐101(Cr)‐NH_2_ film. The overall thicknesses of the films were ≈13 and 16 µm for MIL‐101(Cr) and MIL‐101(Cr)‐NH_2_ films, respectively (Figure S5, Supporting Information). All surface topographic characteristics of the obtained MOF films at the micrometer scale demonstrate their practicability for printing features in the range of tens or hundreds of micrometers on the MIL‐101(Cr) and MIL‐101(Cr)‐NH_2_ film via µCS, making them suitable substrates for microarrays.

### 2.3 Patterning Via µCS

**Figure 4:**
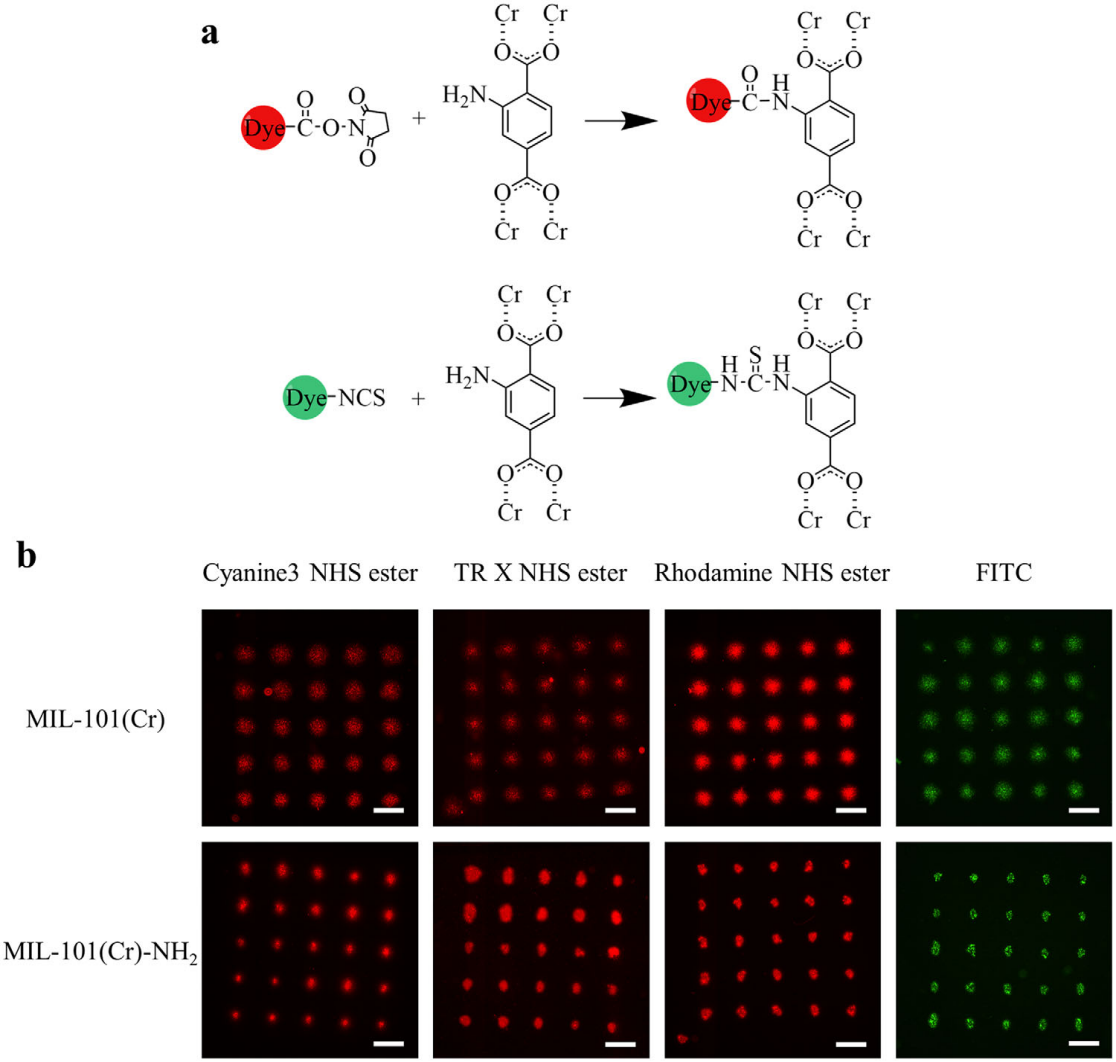
a) Reaction formulas between the amino group on the linker of MOF and the dyes with succinimidyl esters (upper) or isothiocyanates (bottom). b) The fluorescent microarrays of dyes printed on MOF films via µCS. The scale bars represent 250 µm.

Having successfully obtained the MOF thin film substrates, the next step is to introduce the dye into the MOF framework as the fluorescent probe by µCS micropatterning. Fluorescent organic dyes were widely used in fluorescent chemosensors as chromophores due to their intrinsic selectivity and high fluorescence quantum yield.^[^ ^37^ ^]^ Among the various organic dyes, those with red and green emissions, which can be visible easily, such as fluoresceins and rhodamines, have been studied extensively for sensing applications.^[^ ^38^ ^]^ So four derivatives of typical red and green fluorescent dyes with succinimidyl esters or isothiocyanates functional groups that can react with the amino groups on the MOF linker were chosen in this study: texas red X (TR X) n‐hydroxysuccinimide (NHS) ester, rhodamine NHS ester, cyanine3 NHS ester, and fluorescein isothiocyanate (FITC), whose structures are shown in Figure S6 (Supporting Information), and the reaction formulas with amino are shown in **Figure** **4**a. They have different structures or functional groups that may show different affinity to analytes, and their molecular size matches the window size of MIL‐101, allowing them to enter the pore of MOF (Figure S6, Supporting Information). These dyes have been shown to exhibit good fluorescent intensities in MIL‐101(Cr) or MIL‐101(Cr)‐NH_2_ under an inverted fluorescence microscope (Figure S7, Supporting Information). Fluorescence spectra of dyes coated on glass surfaces and incorporated into MOF thin films were recorded to investigate changes in their fluorescence properties, which are shown in Figure S8 (Supporting Information). All spectra were background‐subtracted with the signals from the glass or MOF thin film and then normalized. A red shift of the emission peaks is observed in all spectra of dyes@MOF, indicating the interaction between the dyes and the MOF framework. Additionally, the emission spectra of cyanine3 HNS ester and TR X NHS ester broadened upon incorporation into MIL‐101(Cr) and MIL‐101(Cr)‐NH_2_, further demonstrating alterations in their fluorescence properties. Then, 5 × 5 fluorescent microarrays with a pitch of 300 µm were obtained in the MOF films by subsequently contacting SPT tips loaded with a dye ink with controlled dwell time and humidity. Each spot was repeated several times to ensure the saturated permeation of ink to get the highest intensity of microarrays, and excess ink was washed off after the incubation. As shown in Figure 4b, all dyes were immobilized on the MOF films as microarrays with ideal fluorescence intensity. The dimensions of spots on the MIL‐101(Cr) films are larger than those on the MIL‐101(Cr)‐NH_2_ because of the different wettability of MOF films caused by roughness and crystal arrangement density differences. The wettability of the MOF thin films was characterized by measuring the water contact angle (WCA) at multiple locations on the surface (Figure S9, Supporting Information). The roughness of a surface can affect its wettability, resulting in a higher WCA than that observed on a surface with the same composition but a smoother texture.^[^ ^39^ ^]^ Additionally, chemical heterogeneities on a surface can pin the contact line, thereby limiting the extent of liquid spreading.^[^ ^39^ ^]^ From the WCA images, water droplets rapidly penetrated into the MIL‐101(Cr) film upon contact, while droplets remained on the surface of MIL‐101(Cr)‐NH_2_, exhibiting WCA ranging from 18° to 59°. Consequently, there are minor reductions in the dimensions of four dye spots in the MIL‐101(Cr) films between the images taken after printing and after washing since the excess ink was washed away (Figure S10, Supporting Information and **Table** **1**). The spots on the MIL‐101(Cr)‐NH_2_ films after a 24 h incubation and washing were slightly larger than those just after printing because ink remained on the MIL‐101(Cr)‐NH_2_ film surface as a droplet for some time with the presence of glycerol to slow the evaporation of solvent and the penetration of the ink into the MOF film occurred during the whole printing and incubation process. The center of the spots is brighter, and the intensity of the edges is the weakest because the penetration direction is from the center (where the tip touched) to the surroundings. The above results indicate that fluorescent microarrays can be achieved in the MOF films via µCS. Furthermore, the dimensions and shapes of the spots are designable, as they were unaffected by the washing and incubation steps. As the inks penetrated downward into the MOF thin films during printing and the unbound dye molecules were thoroughly washed away, the resulting 3D distribution of dyes within the films that bound to the framework or impregnated within the porous structure was visualized by confocal microscopy (Figure S11, Supporting Information). The red‐emitting dyes penetrated deeply into the MIL‐101(Cr) films (7.6‐15.2 µm), whereas FITC reached only ≈7.3 µm. In MIL‐101(Cr)‐NH2 films, cyanine3 NHS ester and rhodamine NHS ester exhibited greater penetration depths (23.0 and 21.2 µm, respectively) compared to TR X NHS ester and FITC (12.9 and 14.0 µm, respectively).

**Table 1:** Dimensions of fluorescent spots in MOF films after printing and after washing

	MIL‐101[Cr]		MIL‐101[Cr]‐NH_2_	
	After printing (µm)	After washing (µm)	After printing (µm)	After washing (µm)
Cyanine 3 NHS ester	173.7 ± 9.5 ×156.2 ± 24.3	186.6 ± 15.4 ×147.9 ± 14.8	30.9 ± 3.5 ×49.1 ± 7.1	43.3 ± 5.8 ×62.7 ± 7.0
TR X NHS ester	78.7 ± 13.0 ×91.5 ± 8.9	79.5 ± 12.0 ×85.7 ± 13.9	39.1 ± 6.2 ×49.7 ± 8.2	51.9 ± 10.8 ×72.3 ± 7.0
Rhodamine NHS ester	96.5 ± 17.2 ×92.6 ± 11.6	80.6 ± 10.9 ×82.9 ± 9.3	47.3 ± 7.2 ×81.5 ± 11.3	54.0 ± 5.3 ×83.5 ± 9.9
FITC	71.8 ± 7.2 ×73.4 ± 6.9	53.3 ± 8.1 ×48.9 ± 8.6	28.3 ± 4.8 ×35.5 ± 6.3	34.3 ± 4.9 ×40.0 ± 6.9

**Figure 5:**
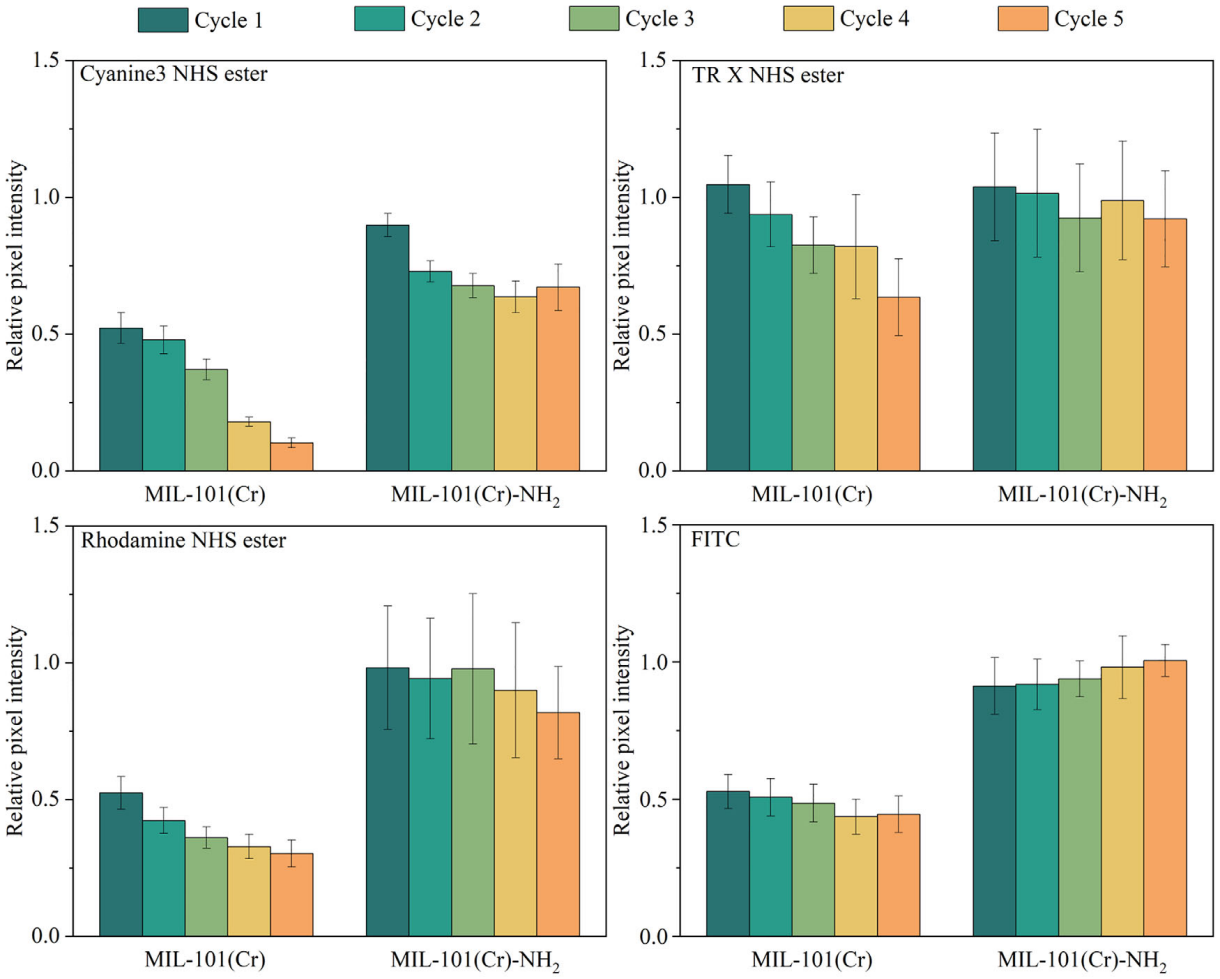
The relative intensity of fluorescent microarrays on the MOF film after repeated 5 rounds of immersion in PBS and washing. Error bars represent the standard deviation. Sample size *n* = 25.

In principle, all dyes were covalently bound to the linker of MIL‐101(Cr)‐NH_2_, while they were immobilized on the MIL‐101(Cr) only by non‐covalent interaction, such as π‐π interaction and the confinement of MOF pores, thus, the bonding strength is weaker, and the dye molecules can leak out easier. Since the fabricated dyes@MIL‐101(Cr) fluorescence microarrays are intended for detection in fluidic environments but are stored under dry conditions prior to use, fluorescence stability during the sensing process, which is primarily influenced by dye leakage from the MOF framework, is critical for their performance. Therefore, dye leakage in phosphate buffered saline (PBS) over the duration of the sensing measurement was investigated. Patterned samples were washed thoroughly with ethanol and water to remove the free excess dye molecules on the surface and then immersed in PBS (pH 7.4) for 15 min to obtain fluorescence images. The immersion time was consistent with the detection measurement duration. Each fluorescence spot's pixel intensity (PI) was measured for the following analysis. The relative PIs of dye@MIL‐101(Cr) and dye@MIL‐101(Cr)‐NH_2_ after each cycle in PBS are illustrated in **Figure** **5**. The intensity of cyanine3, rhodamine NHS ester, and FITC in MIL‐101(Cr) film decreased to ≈50% just after the first cycle. Cyanine3 NHS ester@MIL‐101(Cr) lost ≈90% of its intensity after five cycles, whereas FITC@MIL‐101(Cr) and Rhodamine NHS ester@MIL‐101(Cr) showed little change in intensity in the subsequent cycles, with 44% and 30% of intensity remaining after five cycles, respectively. The intensity of TR X NHS ester@MIL‐101(Cr) was the most stable compared to the other three, eventually decreasing by only 37%. For MIL‐101(Cr)‐NH_2_, the covalent bonding greatly encouraged the stability of the dye@MIL‐101(Cr)‐NH_2_ composites in solution. After five cycles, the intensity of FITC@MIL‐101(Cr)‐NH_2_ remained almost unchanged, and the leakage of TRX NHS in the MIL‐101(Cr)‐NH_2_ film was negligible, dropping by only 8%. Although the intensities of the other two dye@MIL‐101(Cr)‐NH_2_ composites still decreased slightly during cycling, they gradually stabilized without significant changes between the two cycles. All the results indicate that the affinities of dyes toward MIL‐101(Cr) are insufficient to maintain a stable binding state in the solution, which would affect the accuracy of sensing results, while the leakages of dyes in the MIL‐101(Cr)‐NH_2_ films are within the acceptable range to work in the fluidic environment, especially after initial conditioning to remove remaining unbound dye molecules in a first PBS cycle. Furthermore, the long‐term stability of the fluorescence probes in solution is also crucial for their practical application in liquid detection. To evaluate this, the dyes@MIL‐101(Cr)‐NH_2_ microarrays were exposed to PBS for five days following thorough washing to remove unbound dyes, and their fluorescence intensity was recorded daily. The results are shown in Figure S12 (Supporting Information), and all dyes@MIL‐101(Cr)‐NH_2_ composites exhibited negligible changes in fluorescence intensity over the five‐day period. These results suggest that the printed dyes@MIL‐101(Cr)‐NH_2_ fluorescence microarrays possess promising stability in aqueous environments, supporting their potential for reliable and extended use in fluidic sample analysis.

### 2.4 pH Sensitivity and Disease‐Associated Molecules Detection in Buffer

**Figure 6:**
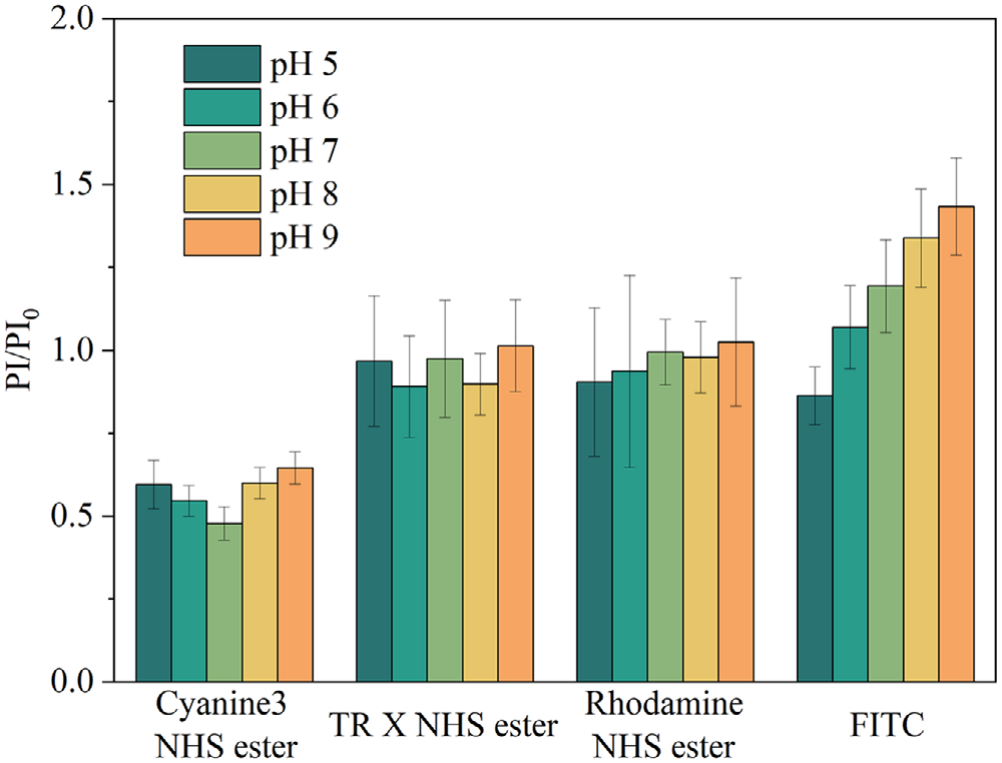
pH sensitivity of dyes@MIL‐101(Cr)‐NH_2_. Error bars represent the standard deviation. Sample size *n* = 16.

The physiological pH of biofluids remains constant in the body and is regulated by metabolism and pulmonary ventilation, and is therefore one of the factors to evaluate health conditions.^[^ ^40^ ^]^ Since FITC exhibits a pH‐dependence fluorescence emission in the pH 5–9 range, many researchers have utilized FITC or its composite as a pH probe in biological conditions, such as in living cells.^[^ ^41^, ^42^ ^]^ In contrast, rhodamine, cyanine3, and TR were demonstrated as pH insensitive under physiological pH conditions.^[^ ^43^, ^44^, ^45^ ^]^ Hence, the pH sensitivity of dyes@MIL‐101(Cr)‐NH_2_ at the pH 5–9 range was also studied. For each sample, the PIs were measured in water as *PI_0_* and in PBS with different pHs as *PI*. The ratios of *PI* and *PI_0_* were used for comparison to eliminate the influence of different initial fluorescence intensities between samples. The fluorescence intensity of FITC@MIL‐101(Cr)‐NH_2_ increased as the pH rose from 5 to 9 (**Figure** **6**), consistent with the pH‐dependent trend of FITC reported in literature, while others showed no apparent change in intensity, suggesting that the combination between dyes and MOF did not change the pH selectivities of dyes and FITC@MIL‐101(Cr)‐NH_2_ can work as a pH probe for biofluid sample in the pH 5–9 while the other three can be incorporated into the array‐based sensing as references without pH sensitivity.

**Figure 7:**
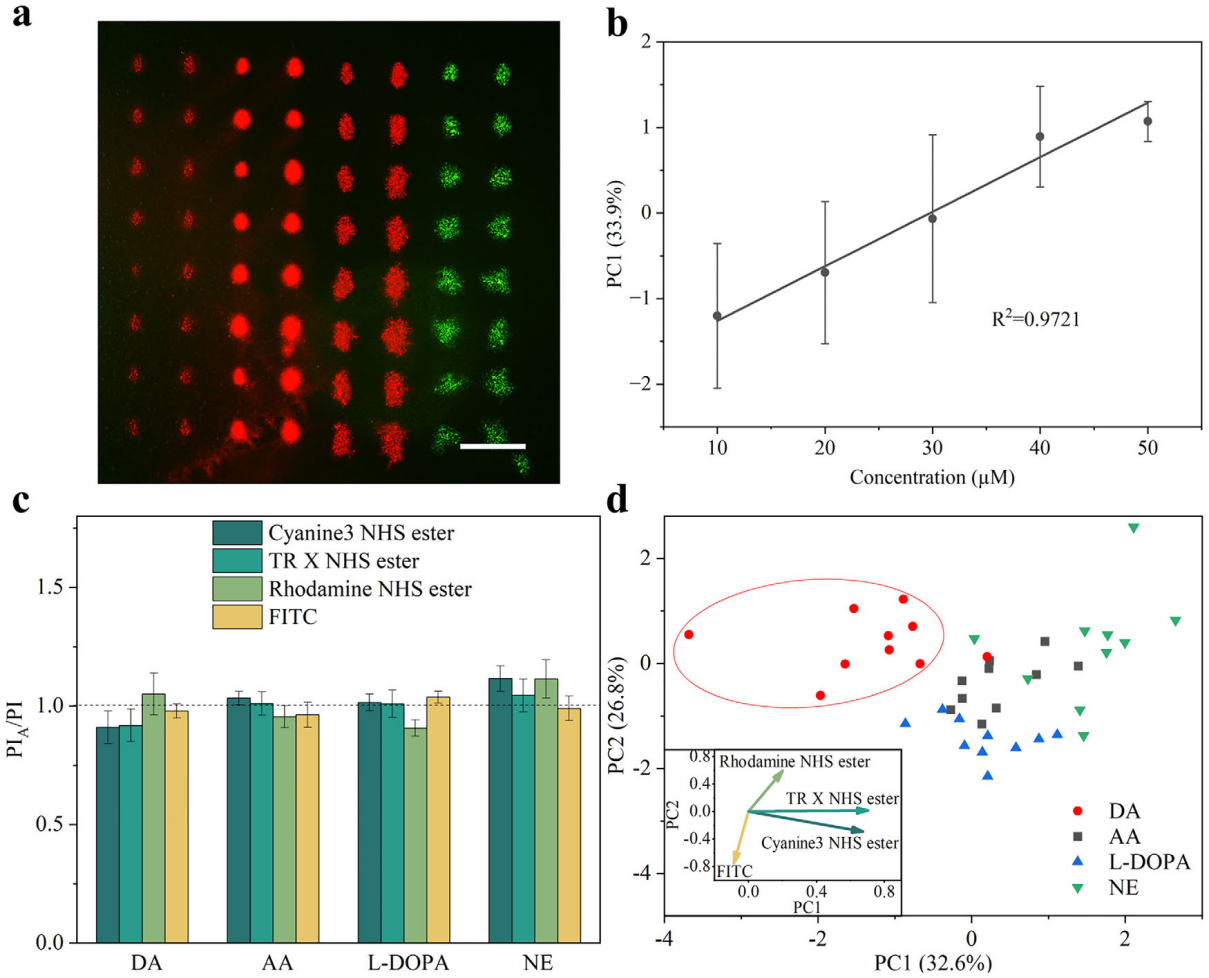
a) Fluorescent image of dyes@MIL‐101(Cr)‐NH_2_ fluorescent sensing array.TR X NHS ester, Cyanine3 NHS ester, Rhodamine NHS ester, and FITC in order from left to right. The scale bars represent 250 µm. b) The linear relationship between the PC1 and the concentration of DA in a range of 10–50 µm. Error bars represent the standard deviation. Sample size *n* = 16. c) Fluorescent intensity changes of dyes@MIL‐101(Cr)‐NH_2_ microarrays after the addition of the analyte. Error bars represent the standard deviation. Sample size *n* = 16. d) 2D PCA plot for the analysis of 50 µm DA, AA, L‐DOPA, and NE.

Dopamine (DA) is a neurotransmitter that plays an essential role in the nervous, cardiovascular, and hormonal systems, as its abnormal levels are often associated with some diseases, such as hypertension, depression, Parkinson's disease, and schizophrenia.^[^ ^46^, ^47^, ^48^, ^49^ ^]^ Therefore, the detection of DA in biofluids can facilitate understanding the physical condition and discovering the related nervous and cardiovascular diseases early in their outsets, and many efforts have been devoted to the specific recognition of DA as it is always present along with its analogues and some potential interferents in biological samples, complicating its detection.^[^ ^50^ ^]^ FITC shows its capacity to detect DA in solution due to the specific interaction between the isothiocyanate group of FITC and the NH_2_ group of DA, as well as the H‐binding and π‐π stacking between FITC and DA molecules.^[^ ^51^ ^]^ These interactions resulted in a change in the fluorescence intensity of FITC. Thomas et al. demonstrated the electrostatic interaction between rhodamine B and DA, which can potentially enhance the sensitivity of DA.^[^ ^52^ ^]^ Therefore, in this study, DA was chosen as the target analyte, and the quantitative detection capability of dyes@MIL‐101(Cr)‐NH_2_ toward DA was investigated using array‐based sensing in a microfluidic channel system. A fluorescent sensing array based on dyes@MIL‐101(Cr)‐NH_2_ for DA detection is shown in **Figure** **7**a. Fluorescence measurements were performed in PBS containing varying concentrations of DA, and the PI of each microarray spot was recorded as an individual readout. Here, *PI_A_* and *PI* represent the PI of dyes@MIL‐101(Cr)‐NH_2_ in the presence and absence of the analyte, respectively, and the *PI_A_*/*PI* values of the four composites were further analyzed using PCA. As shown in Figure 7b, the first principal component (PC1) obtained from the PCA results exhibited a linear relationship with DA concentrations in the range of 10–50 µm, with a limit of detection (LOD) of 9.19 µm and a limit of quantification (LOQ) of 27.86 µm, demonstrating the capability of dyes@MIL‐101(Cr)‐NH_2_ for quantitative detection of DA in the micromolar range. FITC@MIL‐101(Cr)‐NH_2_ has been demonstrated to be pH‐sensitive, while DA contains one primary amino group and two phenolic hydroxyl groups, all of which exhibit pH‐dependent H‐binding behavior in the solution^[^ ^53^ ^]^ In addition, the electrostatic interactions between MOF composites and analytes also vary with pH.^[^ ^54^ ^]^ Therefore, the fluorescence response of FITC@MIL‐101(Cr)‐NH_2_ upon exposure to 50 µm DA at different pH values is shown in Figure S13 (Supporting Information). As the fluorescence response may be governed by multiple noncovalent interactions between FITC@MIL‐101(Cr)‐NH_2_ and DA, including H‐binding and electrostatic interactions, and the H‐binding is not the primary driving force, no clear correlation is observed between changes in fluorescence intensity and pH. The consistent response to DA across different pH also indicates the functional stability of FITC@MIL‐101(Cr)‐NH_2_ within the pH range of 5 to 9. Furthermore, a discrimination test was performed using spiked solutions with individual analytes, including DA and potential interferents: ascorbic acid (AA), levodopa (L‐DOPA), and norepinephrine (NE). Under physiological conditions, the concentration of DA in nervous and bodily fluids typically ranges from 0.01 to 1 µm.^[^ ^55^ ^]^ In elderly patients with Alzheimer's disease, plasma DA levels have been reported to reach up to 18 µm.^[^ ^56^ ^]^ NE and L‐DOPA are present in blood at much lower concentrations, ranging from 0.001 to 0.007 µm, while AA is found at significantly higher concentrations than DA within the range of 5 to 100 µm.^[^ ^57^, ^58^ ^]^ However, considering the LOD and LOQ of dyes@MIL‐101(Cr)‐NH_2_ microarrays, a relatively high analyte concentration of 50 µm was selected to provide preliminary evidence of their discrimination capability. While the current setup is therefore could be of direct use for screening biofluids directly for pathophysiological concentrations of DA, measuring physiological concentrations would require pre‐concentration of the sample liquid, e.g. by partly evaporation of water or full lyophilization and resuspension. Compared to the enzymes and antibody sensors with highly specific recognition, the dyes@MIL‐101(Cr)‐NH_2_ fluorescent chemosensors only have partial selectivity due to the weaker non‐covalent interaction between dyes and DA, but they provided different fingerprints of fluorescent response for different analytes in Figure 7c. The fluorescent intensity response data *PI_A_/PI* were visualized by 2D and 3D PCA (Figure 7d; Video S3, Supporting Information, respectively). In the PCA plots, there was no overlap between 90% of the data for DA and the other interferences, suggesting that it is possible to distinguish DA from AA, L‐DOPA, and NE by the dyes@MIL‐101(Cr)‐NH_2_ already with standard dyes to a surprisingly high degree. To identify which dyes@MIL‐101(Cr)‐NH_2_ in the fluorescence microarrays are best suited for discriminating the analytes, the loading plot of the PCA, showing the correlation coefficients between principal components and the sensor elements within the fluorescence arrays, is presented as the inset in Figure 7d. Cyanine3 NHS ester and TR X NHS ester exhibit strong positive loadings on the PC1, while cyanine3 NHS ester also shows a slight negative loading on the second principal component (PC2). As a result, these two dyes responded more strongly to analytes positioned in the positive region of PC1, such as AA, L‐DOPA, and NE. Rhodamine NHS ester shows a strong positive loading on PC2, while FITC has a negative loading on PC1, indicating that they contributed more to the detection of DA, whose data points are primarily located in the negative region of PC1 and the positive region of PC2. In addition to the dyes and MOF framework used in this study, other reasonable combinations of dyes and MOF can also be achieved by this strategy. Hence, further studies could focus on the design of different dyes@MOF composites with higher sensitivity and selectivity toward disease‐associated molecules and their application in a real biofluid sample.

## 3 Conclusion

To summarize, the present study set out to pattern the fluorescent microarrays on MOF thin films, which are fabricated by RMC using a “receding front” idea in evaporation of MOF dispersion to create an in situ “blade” for spreading the thin film and demonstrate their potential for array‐based sensing of small molecule analytes. The MOF thin films obtained via RMC exhibited superior surface morphology, making them suitable for use in the µCS patterning process of dyes@MOF composites, in comparison to those produced by spin coating and drop casting. The dyes@MIL‐101(Cr)‐NH_2_ composites were proved to be stable in solution due to the presence of covalent bonding between dyes and ‐NH_2_ group on the MOF ligands. Four dyes were integrated into one MOF film for multiplexed array‐based sensing in solution, and the FITC@MIL‐101(Cr)‐NH_2_ presented the pH‐dependent emission in the pH range 5–9. The dyes@MIL‐101(Cr)‐NH_2_ microarrays served as a fluorescence probe for the detection of DA, exhibiting a LOD of 9.19 µm and maintaining functional stability over a pH range of 5 to 9. With different fluorescence response behaviors of four dyes@MIL‐101(Cr)‐NH_2_ composites, this sensor showed fingerprints to four metabolites, and dopamine can be distinguished from the other three interferents. These results demonstrate that the RMC is a potential tool for fabricating high‐quality MOF thin film, especially for large areas, and patterning the fluorescent microarrays on the MOF thin films is a powerful technique for the development of novel surface‐based fluorescent MOF detecting systems that can simplify the pre‐treatment of MOF composites in solution, facilitate handling (e.g., by integration in microfluidic devices), lower the error due to the MOF suspension, and reduce the consumption of materials. However, in this demonstration of principle, the chemosensor still has certain limitations in terms of selectivity and sensitivity, and further work should focus on the choice or development of sensing components with higher selectivity and distinct response behaviors, and thus improve the performance of the chemosensors further.

## 4 Experimental Section

### Materials and Methods

Chromium (ΙΙΙ) nitrate nonahydrate (Cr(NO_3_)_3_∙9H_2_O), terephthalic acid (BDC), dopamine hydrochloride (DA), L‐ascorbic acid (AA), levodopa (L‐DOPA), DL‐norepinephrine hydrochloride (NE), fluorescein isothiocyanate isomer I (FITC), sodium hydroxide (NaOH), chloroform, tetrahydrofuran (THF), triethylamine (TEA), dimethyl sulfoxide (DMSO), glycerol and ammonium fluoride (NH_4_F) were purchased from Sigma‐Aldrich (Germany). 2‐Aminoterephthalic acid (NH_2_‐BDC) and rhodamine n‐hydroxysuccinimide (NHS) ester were supplied from Thermo Scientific (USA). Texas red X (TR X) NHS ester and cyanine3 NHS ester were purchased from Lumiprobe (Germany). Ethanol and phosphate buffered saline (PBS) were obtained from VWR (Germany). All the reagents were used without further purification. Distilled water was obtained in the lab from an Arium Pro system, Sartorius (Germany).

### MOF Synthesis

MIL‐101(Cr) with nano‐size was synthesized by HF‐free hydrothermal synthesis as described in the publication with slight modification.^[^ ^27^, ^28^ ^]^ First, 792.297 mg Cr(NO_3_)_3_∙9H_2_O and 328.937 mg BDC were dispersed in 60 mL distilled water and stirred for 1 h to obtain the homogenous mixture, which was then sealed in a Teflon‐lined autoclave (100 mL) and heated in a UFP‐500 oven (Memmert, Germany) for 24 h at 180 °C. After cooling to room temperature, the raw product was filtered with Whatman filter paper (Cytiva, United Kingdom) to remove the recrystallized BDC. Then, the green product was collected by centrifugation (Universal 320 centrifuge, Hettich, Germany) and washed three times with ethanol before drying at room temperature. Next, the obtained solid sample was purified with hot ethanol at 80 °C for 4 h, then separated by centrifugation and washed three times with ethanol before drying at room temperature. The synthesized MIL‐101(Cr) was further purified in an aqueous solution of 30 mm NH_4_F at 60 °C for 5 h to remove the traces of impurity in the pores, then washed five times with hot water. After drying at room temperature, the pure MIL‐101(Cr) was successfully obtained. MIL‐101(Cr)‐NH_2_ was prepared with alkaline assistance following the literature.^[^ ^26^ ^]^ 1600 mg Cr(NO_3_)_3_∙9H_2_O, 720 mg NH_2_‐BDC, and 400 mg NaOH were added to 30 mL of distilled water and stirred for 30 min. The above solution was sealed in a Teflon‐lined autoclave and heated for 12 h at 150 °C. The crude product was washed several times with hot ethanol and then dried at room temperature to get pure MIL‐101(Cr)‐NH_2_.

### MOF Suspension Preparation and Substrate Pre‐Treatment

The MOF powder was dispersed in a solvent to get 5 mg mL^−1^ MIL‐101(Cr) in ethanol and 1 mg mL^−1^ MIL‐101(Cr)‐NH_2_ in THF, and stirred for the homogeneous mixture until use. The glass substrates (18×18 mm microscope cover slips for general experiments or 76 × 26 mm microscope slides used for sensing experiments, from VWR, Germany) used for the MOF thin film fabrication were washed in chloroform, ethanol, and water for 5 min separately under sonication before drying under the stream of nitrogen. Then, the cleaned glass substrates were activated by oxygen plasma for 10 min (10 sccm O_2_, 0.2 mbar, and 100 W, ATTO system, Diener electronics, Germany) just before use.

### Drop Casting

The 20 uL MOF suspension was dropped on the substrate by micropipette and dried at room temperature. After drying thoroughly, the above process was repeated several times until the desired thickness. Then, the dried MOF thin film was heated to 130 °C to make it more stable. Finally, the film was washed with ethanol or THF.

### Spin Coating

The MOF thin films obtained by the spin coating were prepared in the WS‐650HZB‐23NPPB spin coater (Laurell, USA) at 1000 rpm for 30 s before repeating, heating, and washing, which is the same as that of drop coating.

### Receding Meniscus Coating (RMC)

Two cleaned glass substrates were used: one for the cover and a plasma‐treated one for the MOF deposition. Then, the MOF suspension was injected into the gap between the two glass substrates, which were bonded together on the sides with double‐sided adhesive tape. After drying, injections of MOF suspension were repeated several times, and MOF films were obtained on the bottom glass. Finally, the MOF films were heated to 130 °C for 10 min and washed.

### Microchannel Cantilever Spotting

The microarrays on the MOF thin film were obtained by microchannel cantilever spotting (µCS) on an NLP 2000 system (NanoInk, USA) equipped with SPT probes (SPT‐S‐C30S, Bioforce Nanosciences, USA). The probes were treated with oxygen plasma for 2 min before the ink loading to facilitate the ink transfer. The dye ink was prepared in DMSO with 20% glycerol to slow solvent evaporation and with 2% TEA for FITC or 1% TEA for the other three dyes to facilitate the interaction with the amino group. The spotting process was generated under 50% humidity with constant dwell time, such as 0.5s for MIL‐101(Cr) films and 2s for MIL‐101(Cr)‐NH_2_ films, because of the different transfer efficiency of ink to different films. Each spot was repeated 6‐10 times to get a higher fluorescence intensity. After spotting, samples were placed in a chamber with high humidity for 24 h at room temperature to complete the interaction between dye molecules and the MOF framework.

### pH Sensitivity and Analytes Detection

The MOF films patterned with dye molecules were first immersed in water and PBS, pH 7.4, for pH sensitivity measurement and dopamine detection, respectively. After incubation of 15 min, fluorescent images of the samples were captured as a control. Then, the samples were immersed in the PBS solution with different pH or concentrations of analytes for another 15 min to get fluorescence data by inverted fluorescence microscope.

### Characterization Methods

Fourier transform infrared (FT‐IR) spectra were measured using the NicoletTM iS50 spectrometer (Thermo Scientific, USA) equipped with an ATR module. Power X‐ray diffraction (PXRD) measurements were performed on a D8 Advance X‐ray diffractometer (Bruker, USA) with a Cu Kα radiation source (λ = 1.54056 nm). After gold sputtering (10.3 nm) in the 108auto sputter coater (Cressington, UK), scanning electron microscopy (SEM) images were collected by Leo Gemini 1530 scanning electron microscope (Carl Zeiss, Germany) at 10kV. The N_2_ adsorption isotherms at 77 K were carried out on a 3Flex adsorption analyzer (Micromeritics, USA) after the degassing of MOF samples at 150 °C for 6 h. The surface areas were calculated by the BET method in the relative pressure P/P_0_ range of 0.05–0.25. The WCA was recorded on an OCA‐20 contact angle analyzer (DataPhysics, Germany), 1 µL droplet was dispersed on the MOF film during the measurements. Four random positions of each sample were recorded. 3D morphology analyses were conducted using a Profilm3D optical profilometer (Filmetrics, USA) with white light interferometry (WLI). Fluorescent images were captured by ECLIPSE Ti2‐E inverted microscope (Nikon, Japan). The GIWAXS data were acquired with a Bruker D8 Advance (primary track: unpolarized Cu K‐alpha X‐ray source (40 kV, 40 mA), Goebel mirror, 0.5 mm micromask, 0.3 mm snout; secondary track: DECTRIS Eiger2 R 500 2D detector, sample‐to‐detector distance: 118.1 mm). A Grazing‐incidence angle of 0.2° and an exposure time of 9 h were employed for measurement. GIWAXS Detector images acquired from multiple angles were stitched together based on a projection method, resulting in a combined image as if taken by a vertically aligned detector, and further image processing and analysis were performed utilizing the GIXSGUI MATLAB toolboxes.^[^ ^32^, ^59^ ^]^ The fluorescent spectra were collected using the same microscope equipped with an Avaspec‐2048 spectrometer (Avantes, Netherlands). The spectra were processed by background subtraction, followed by smoothing and normalization. 3D confocal fluorescence microscopy images were recorded using the Leica Stellaris 5 confocal laser scanning microscope (Leica Microsystems, Germany), with post‐processing including background noise reduction and high‐pass edge enhancement.

### Statistical Analysis

The sizes of fluorescent spots were measured by ImageJ 1.53t on 5 × 5 fluorescent microarrays, with each spot considered as a single readout, and fluorescent images were processed by Python 3.9 to read out the pixel intensity. The fluorescence stability results were obtained from 5 × 5 fluorescent microarrays (sample size *n* = 25), while pH sensitivity and detection data were analyzed from 8 × 8 multiplexed fluorescent microarrays (Figure 7a, sample size *n* = 16). All the results were reported as mean ± standard deviation. The randomized design was applied in the PCA analysis with 10 sets of data, and each set of data contained four randomly selected data, which came from four different dye fluorescent microarrays on the same sample. The 2D and 3D PCA results were calculated using Python 3.9.

## Conflict of Interest

The authors declare no conflict of interest.

## Supporting information



Supporting Information

Supplemental Video 1

Supplemental Video 2

Supplemental Video 3

## Data Availability

The data that support the findings of this study are available from the corresponding author upon reasonable request.
