# Supplementary material for: Multiplexed Fluorescent Microarrays on MIL‐101(Cr) Thin Films as Luminescent Probes for pH and Disease‐Associated Molecules
Source: Small. 2025 Sep 10;21(43):e04783. doi: 10.1002/smll.202504783 (PMC12571215; doi:10.1002/smll.202504783)
Supplement: Supplementary file 1 — Supporting Information [file SMLL-21-e04783-s002.pdf]

Supporting Information

Multiplexed Fluorescent Microarrays on MIL-101(Cr) Thin Films as Luminescent Probes for pH and Disease-Associated Molecules

*Wenjing Wang, Wenwu Yang, Maike Schliephake, Tonghan Zhao, Yan Liu, Navid Hussain, Ben Breitung, Andreas H. Schäfer, Pavel A. Levkin, Jasmin Aghassi-Hagmann, Annie K. Powell, Michael Hirtz**

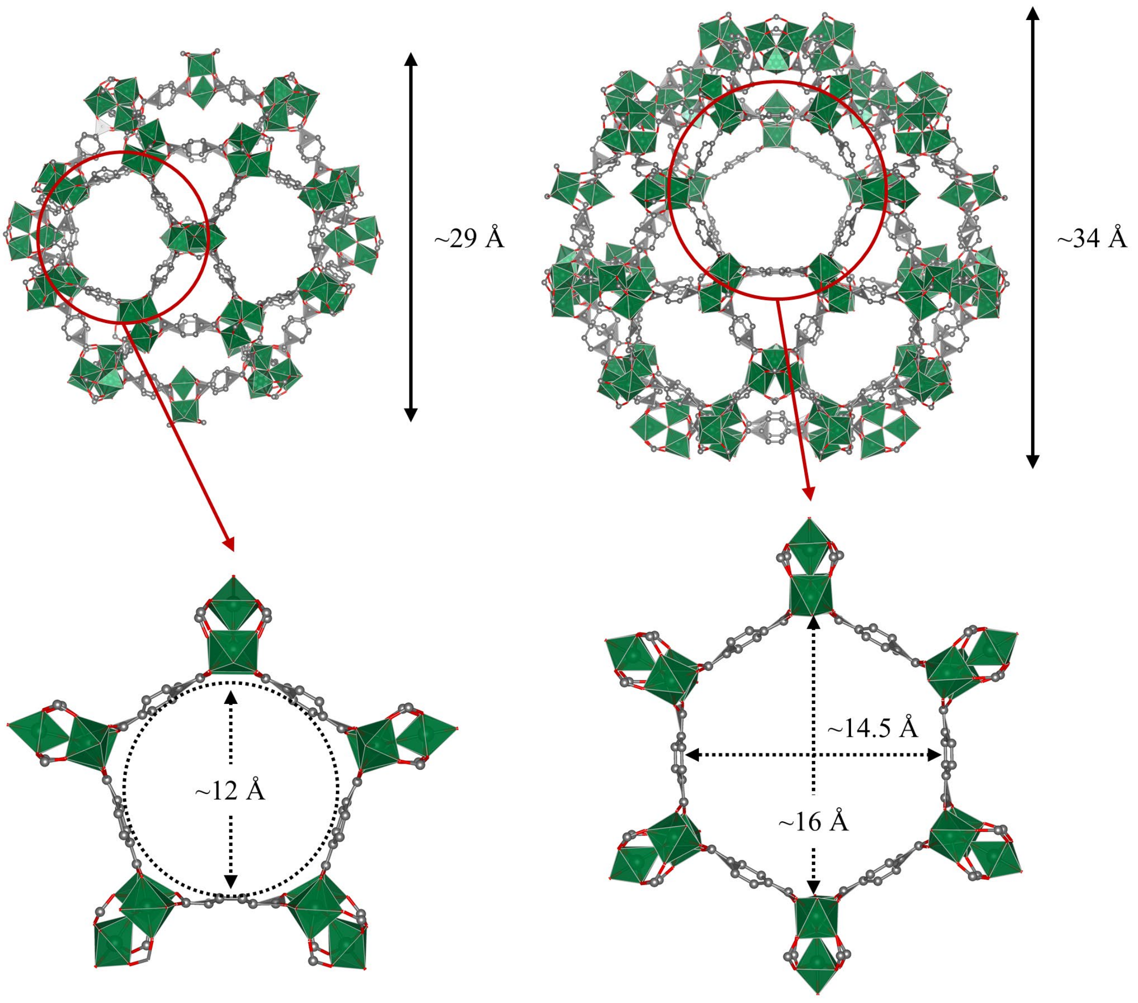

Figure S1. Dimensions of two cages and windows in MIL-101(Cr) structure. Chromium octahedra, oxygen, and carbon atoms are in green, red, and grey, respectively.

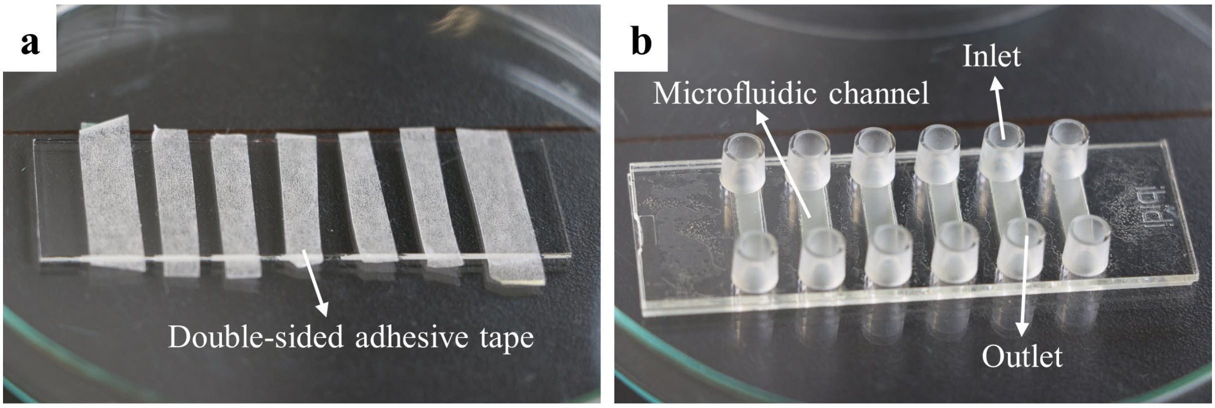

Figure S2. (a) The photograph of a glass substrate with double-sided tape used to fabricate a MOF thin film that can be incorporated into a microfluidic channel system. The area where the MOF thin film is formed is confined between the two strips of tape. (b) The photograph of microfluidic channels with MOF thin films on the bottom.

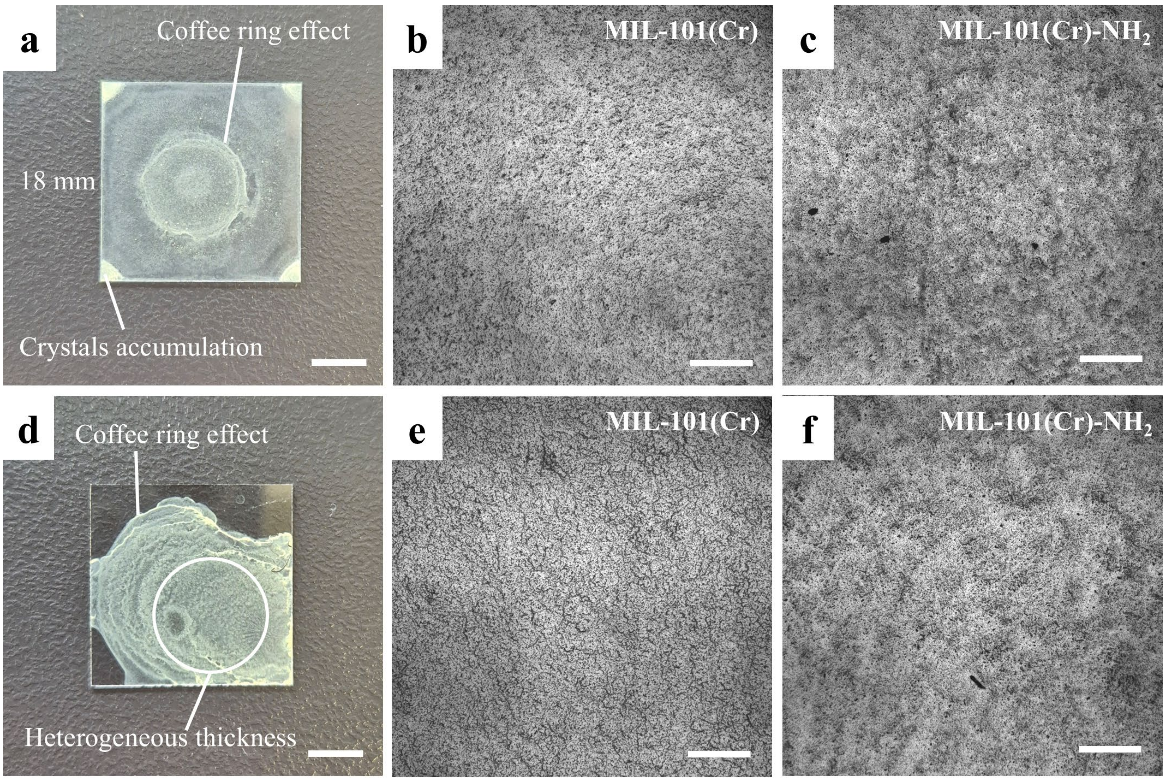

Figure S3. Photographs of MOF film made by (a) spinning coating and (d) drop casting. SEM images of (b) MIL-101(Cr) film and (c) MIL-101(Cr)-NH_2 film made by spinning coating. SEM images of (e) MIL-101(Cr) film and (f) MIL-101(Cr)-NH_2 film made by drop casting. The scale bars represent 5 mm in Photographs and 500 μm in SEM images.

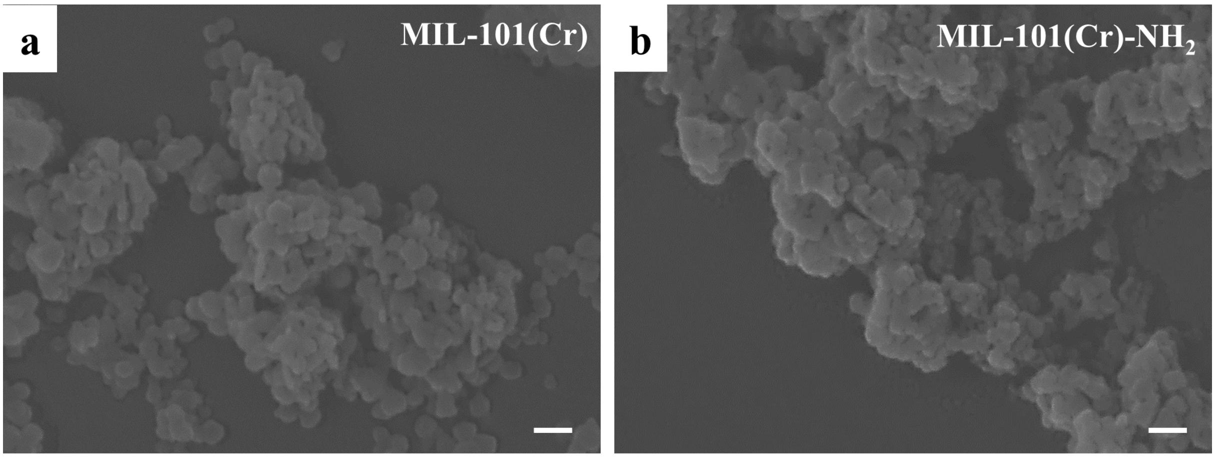

Figure S4. SEM images of (a) MIL-101(Cr) film and (b) MIL-101(Cr)-NH2 film in 70,000× amplification. The scale bars represent 0.1 μm.

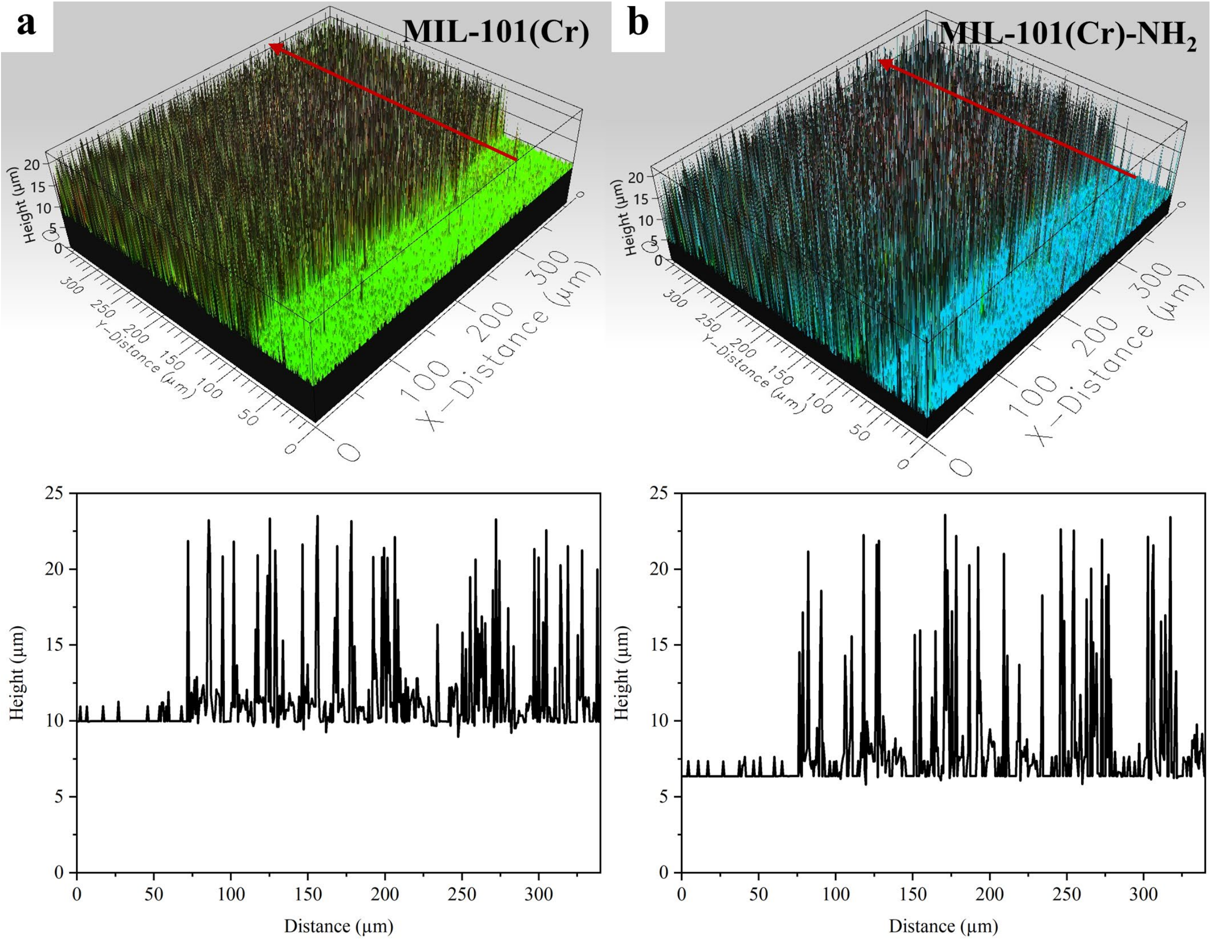

Figure S5. 3D surface morphology of (a) MIL-101(Cr) and (b) MIL-101(Cr)-NH_2 films with height profile of arrow direction.

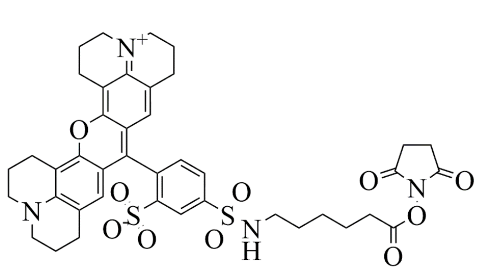

1.98 nm × 1.19 nm ×0.91 nm

TR X NHS ester

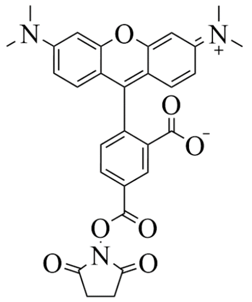

1.62 nm × 1.34 nm ×0.60 nm

Rhodamine NHS ester

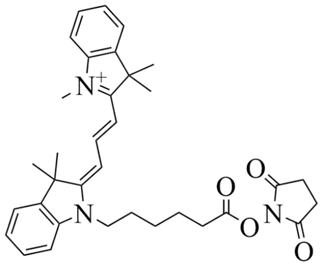

1.93 nm × 1.10 nm ×0.88 nm

Cyanine3 NHS ester

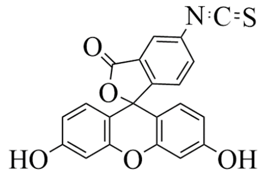

1.05 nm × 0.93 nm ×0.70 nm

FITC

Figure S6. Chemical structures and molecular sizes of texas red X (TR X) n-hydroxysuccinimide (NHS) ester, rhodamine NHS ester, cyanine3 NHS ester, and fluorescein isothiocyanate (FITC). The molecular sizes were calculated by the open-source RDKit toolkit.[1]

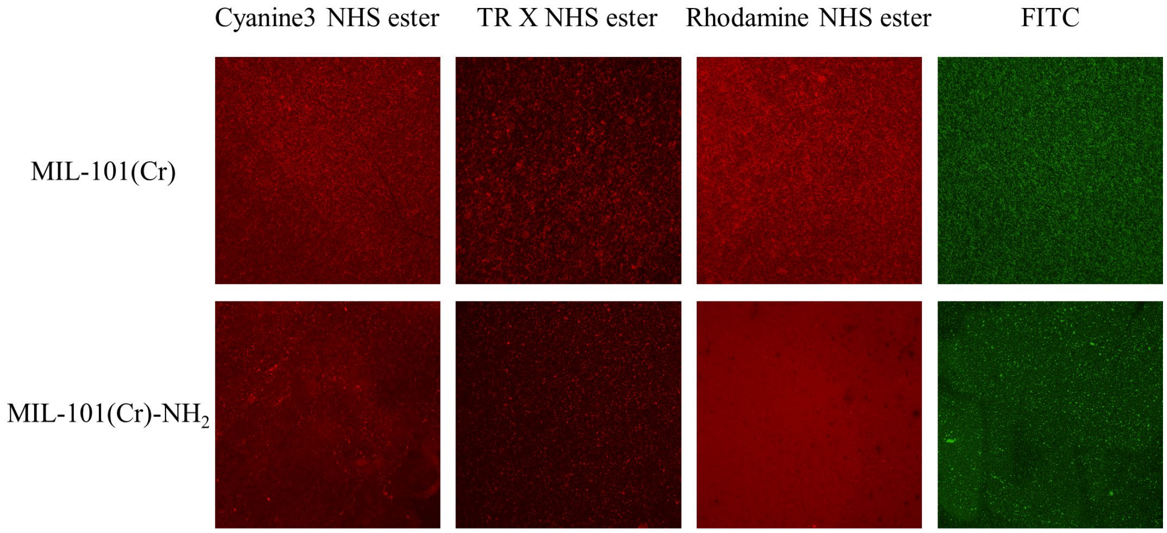

Figure S7. Fluorescent image of dyes@MIL-101(Cr) and dyes@MIL-101(Cr)-NH_2.

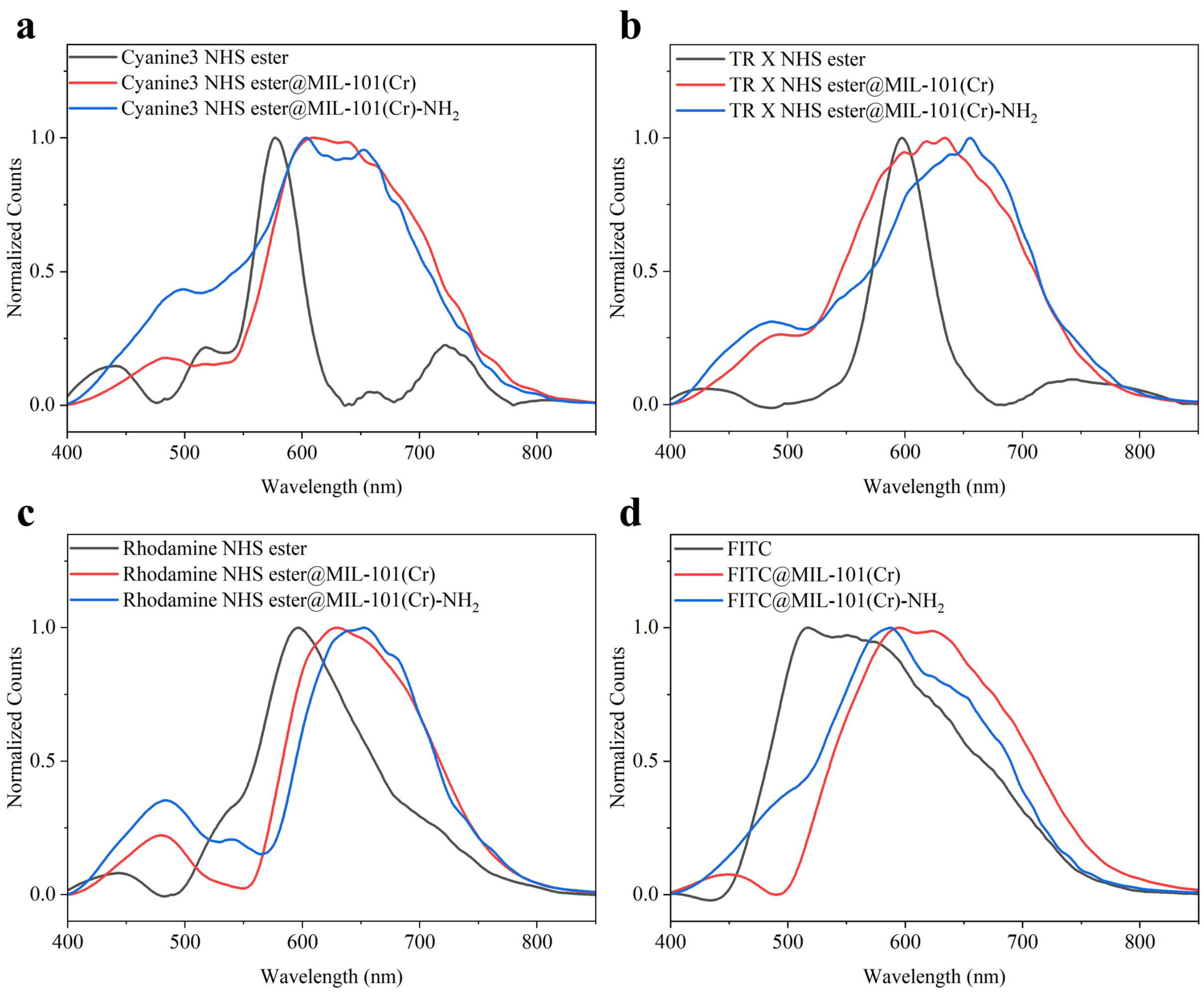

Figure S8. Fluorescence spectra of the dyes and dyes@MIL-101.

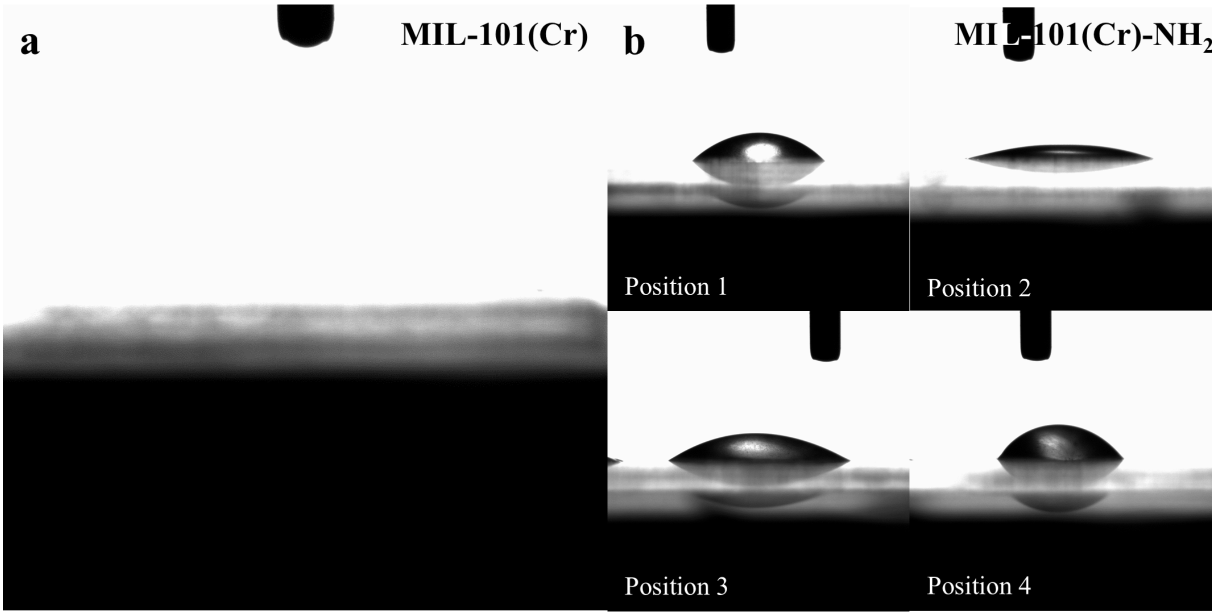

Figure S9. Water contact angle of (a) MIL-101(Cr) film and (b) MIL-101(Cr)-NH_2 film.

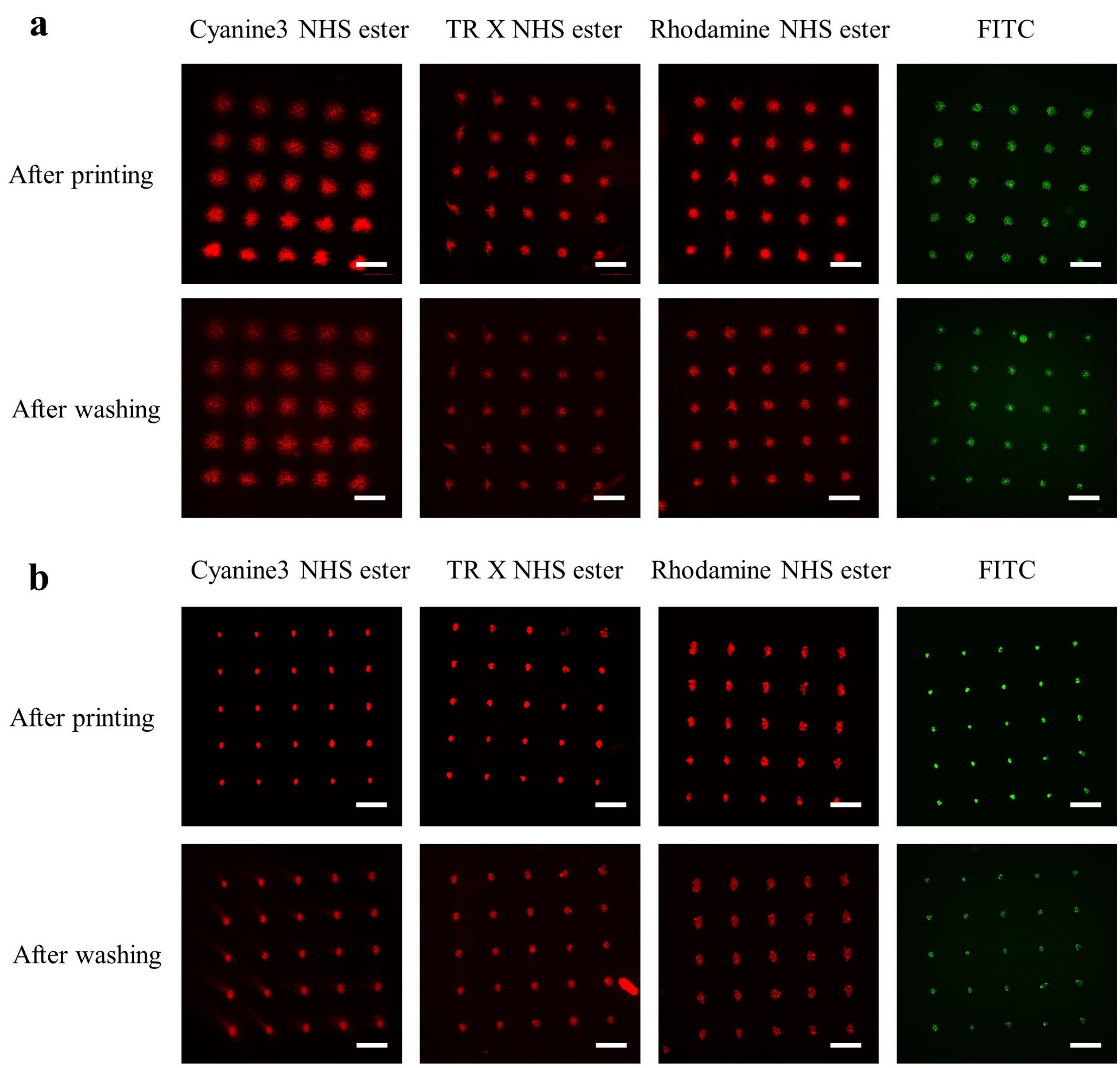

Figure S10. Diameters of microarray spots after printing and after washing for (a) MIL-101(Cr) films and (b) MIL-101(Cr)-NH_2 films. The scale bars present to 250 μm.

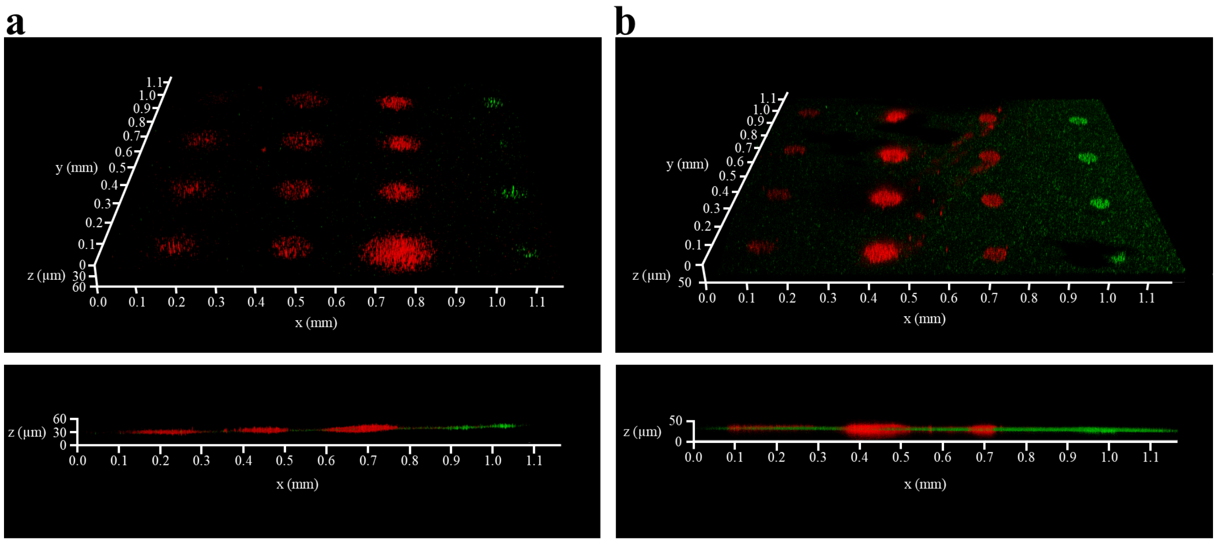

Figure S11. 3D confocal fluorescence microscopy images of microarrays on (a) MIL-101(Cr) and (b) MIL-101(Cr)-NH2 thin films, including cross-sectional views along a line of dots. Dyes from left to right are: TR X NHS ester, cyanine3 NHS ester, rhodamine NHS ester, and FITC.

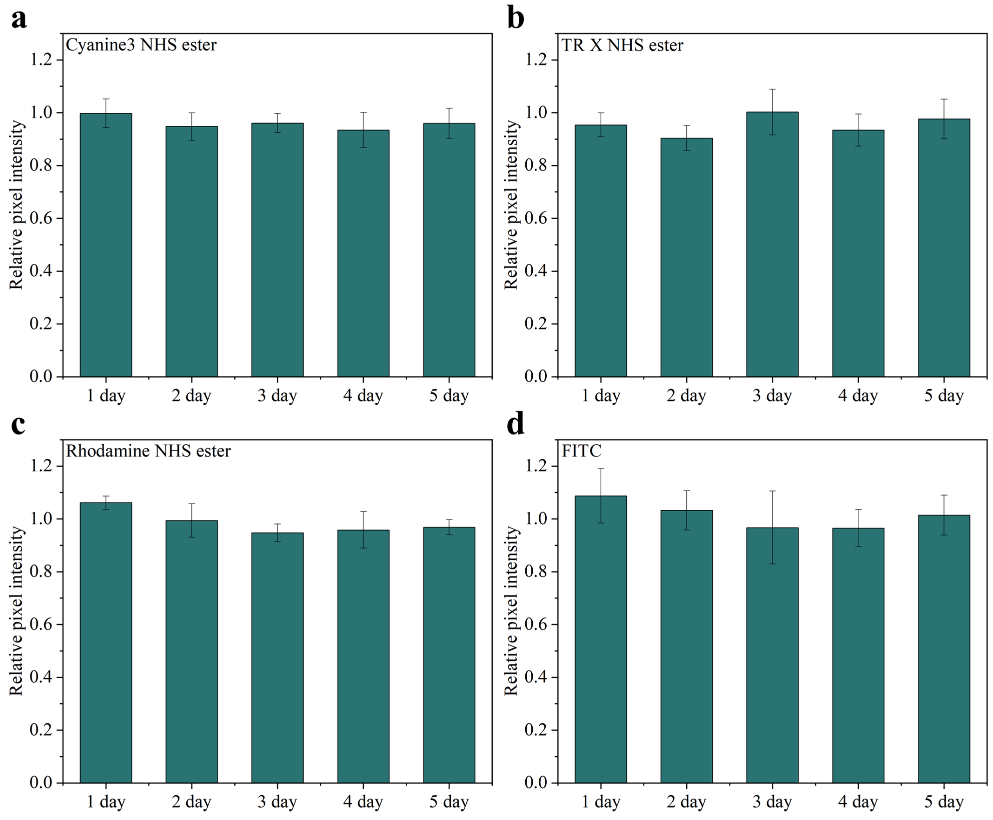

Figure S12. Changes in fluorescence intensity of dyes@MIL-101(Cr)-NH_2 microarrays after five days of exposure in PBS. Error bars represent the standard deviation. Sample size n=10.

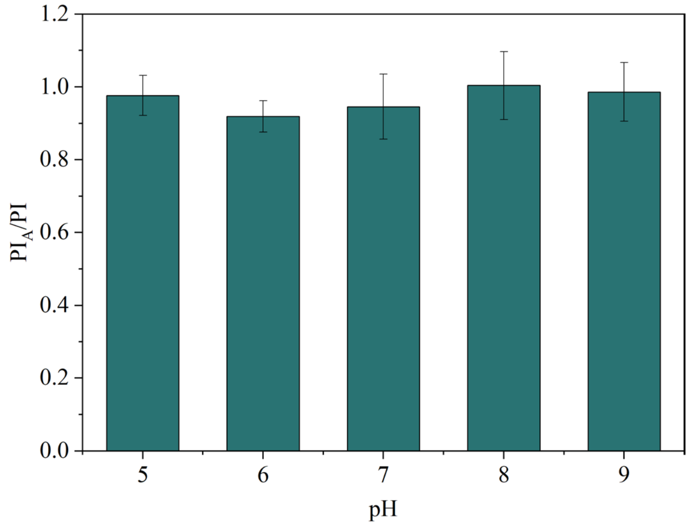

Figure S13. Fluorescence response of FITC@MIL-101(Cr)-NH_2 upon exposure to 50 μM DA at a pH range of 5-9. Error bars represent the standard deviation. Sample size n=10.

REFERENCES

[1] RDKit: Open-Source Cheminformatics Software. https://www.rdkit.org/.
